# Artificial intelligence-driven identification and mechanistic exploration of synergistic anti-breast cancer compound combinations from *Prunella vulgaris* L.-*Taraxacum mongolicum* Hand.-Mazz. herb pair

**DOI:** 10.3389/fphar.2024.1522787

**Published:** 2025-01-07

**Authors:** Chunlai Feng, Jiaxi Cheng, Mengqiu Sun, Chunxue Qiao, Qiuqi Feng, Naying Fang, Yingying Ge, Mengjie Rui

**Affiliations:** School of Pharmacy, Jiangsu University, Zhenjiang, China

**Keywords:** artificial intelligence, traditional Chinese medicine, breast cancer treatment, synergistic combination, *Prunella vulgaris* L., *Taraxacum mongolicum* Hand.-Mazz.

## Abstract

**Introduction:**

The *Prunella vulgaris* L. (PVL) and *Taraxacum mongolicum* Hand.-Mazz. (TH) herb pair, which is commonly used in traditional Chinese medicine (TCM), has been applied for the treatment of breast cancer. Although its efficacy is validated, the synergistic anti-breast cancer compound combinations within this herb pair and their underlying mechanisms of action remain unclear.

**Methods:**

This study aimed to identify and validate synergistic anti-breast cancer compound combinations within the PVL-TH pair using large-scale biomedical data, artificial intelligence and experimental methods. The first step was to investigate the anti-breast cancer effects of various PVL and TH extracts using *in vitro* cellular assays to identify the most effective superior extracts. These superior extracts were subjected to liquid chromatography-mass spectrometry (LC-MS) analysis to identify their constituent compounds. A deep learning-based prediction model, DeepMDS, was applied to predict synergistic anti-breast cancer multi-compound combinations. These predicted combinations were experimentally validated for their anti-breast cancer effects at actual content ratios found in the extracts. Preliminary bioinformatics analyses were conducted to explore the mechanisms of action of these superior combinations. We also compared the anti-breast cancer effects of superior extracts from different geographical origins and analyzed the contents of compounds to assess their representation of the anti-tumor effect of the corresponding TCM.

**Results:**

The results revealed that LC-MS analysis identified 27 and 21 compounds in the superior extracts (50% ethanol extracts) of PVL and TH, respectively. Based on these compounds, DeepMDS model predicted synergistic anti-breast cancer compound combinations such as F973 (caffeic acid, rosmarinic acid, p-coumaric acid, and esculetin), T271 (chlorogenic acid, cichoric acid, and caffeic acid), and T1685 (chlorogenic acid, rosmarinic acid, and scopoletin) from single PVL, single TH and PVL-TH herb pair, respectively. These combinations, at their actual concentrations in extracts, demonstrated superior anti-breast cancer activity compared to the corresponding extracts. The bioinformatics analysis revealed that these compounds could regulate tumor-related pathways synergistically, inhibiting tumor cell growth, inducing cell apoptosis, and blocking cell cycle progression. Furthermore, the concentration ratio and total content of compounds in F973 and T271 were closely associated with their anti-breast cancer effects in extracts from various geographical origins. The compound combination T1685 could represent the synergistic anti-breast cancer effects of the PVL-TH pair.

**Discussion:**

This study provides insights into exploring the representative synergistic anti-breast cancer compound combinations within the complex TCM.

## 1 Introduction

The rising prevalence of breast cancer presents a significant global health challenge (Hong and Xu, 2022). Current treatments, such as surgery, radiotherapy, and chemotherapy, often lead to severe side effects and the development of drug resistance (Pace and Shulman, 2016). As a result, there is growing interest in synergistic anti-breast cancer compound combinations from Traditional Chinese medicine (TCM), which have the potential to overcome drug-induced resistance and minimize drug toxicity (Jin et al., 2018; Zhou et al., 2023; Yu et al., 2024). These combinations work synergistically through multiple compounds, targets, and pathways to exert their effects (Gezici and Şekeroğlu, 2019; Wang et al., 2023; Gao et al., 2024). However, due to the vast number of TCM compounds and their combinations, identifying the most effective synergistic combinations of anti-breast cancer compounds within TCM remains a challenge (Hou et al., 2019).

Traditional experimental approaches for assessing the synergistic compound combinations are often time-consuming and resource-intensive (MacGowan et al., 1990; Sopirala et al., 2010). For instance, Jaaks et al. (2022) screened 2025 drug pair combinations against 125 cancer cell lines using the Genomics of Drug Sensitivity in Cancer (GDSC) database. They identified only one combination of TOP1 and CHEK1 inhibitors that exhibited a synergistic anti-tumor effect *in vivo* (Jaaks et al., 2022). In contrast, there has been a growing interest in the prediction of anti-tumor compound combinations through computational approaches, including machine learning and deep learning (Bansal et al., 2014; Chen et al., 2018; Kuru et al., 2022). These techniques, especially deep learning, have shown superior capabilities in extracting key features and learning patterns from large-scale biomedical data, significantly outperforming traditional models (Baptista et al., 2021; Fan et al., 2021). For instance, DeepSynergy (Preuer et al., 2018) and AuDNNsynergy (Zhang et al., 2021) utilized integrated chemical and genomic data to predict the synergy scores of compound combinations for various cancers. Another deep learning model is DrugCell, an interpretable model trained with data from the response of 1,235 tumor cells to 684 drugs. It could predict treatment responses of human cancer cells and design effective synergistic compound combinations to improve treatment outcomes (Kuenzi et al., 2020).

Current computational approaches primarily focus on predicting compound pairs, so neglecting the difficulty of screening synergetic multi-compound combinations, particularly in TCM. In light of recent advancements in deep learning, we developed DeepMDS (She et al., 2022), a deep neural network designed to predict synergistic multi-compound combinations susceptible to a specific molecular subtype of cancer cells. DeepMDS integrated gene expression profiles of cancer cell lines, target information, and drug response data to predict pseudo-IC_50_ values, which served as indicators for the synergistic anti-tumor effects of compound combinations.

*Prunella vulgaris* L. (PVL) is a perennial herb from the Labiatae family, and its dried ear was called Xiakucao in China. PVL was known for its broad range of pharmacological actions, including anti-tumor (Kim et al., 2012), anti-oxidant (Chen et al., 2019), anti-inflammatory and immunological regulatory effects (Guo et al., 2021). Recent pharmacological studies have provided evidence of the anti-breast cancer effect of PVL, which has been extensively employed in the treatment of breast cancer (Gao and Xu, 2019; Luo et al., 2022). *Taraxacum mongolicum* Hand.-Mazz. (TH), known as Pugongying in China, is a perennial herb in Asteraceae family. It has been widely used to prevent and treat various inflammatory and infectious diseases (Ge et al., 2021). Investigations showed that TH has potential in treating various types of cancer, including breast cancer (Wang et al., 2022), gastric cancer (Zhu et al., 2017), and non-small cell lung cancer (Kang et al., 2021). Together, the PVL-TH pair is traditionally used for its heat-clearing and toxin-removing properties (National Pharmacopoeia Commission, 2020). According to the Collected Works of Materia Medica, the PVL-TH pair combined with wine can be used to treat early acute mastitis. The previous results of animal experiment demonstrated that PVL-TH increased the level of interleukin-6 (IL-6) in breast cancer model mice, inhibiting the growth and promoting the apoptosis of breast cancer cells (Tang et al., 2020). The anti-tumor activities of the PVL-TH pair are attributed to its bioactive compounds. For instance, rosmarinic acid increases the level of apoptotic markers P53 and Caspase-3 and reduces the Bcl2/Bax ratio (Mahmoud et al., 2021). Luteolin suppresses the proliferation of breast cancer cells by elevating the expression of miR-203 and inhibiting Ras/Raf/MEK/ERK signaling (Gao et al., 2019). However, the task of identifying the most effective synergistic combinations of anti-breast cancer compounds from PVL-TH pair remains challenging.

In this study, we used PVL and TH as modeling medicine and integrated artificial intelligence technology with large-scale biomedical data to identify synergistic compound combinations against breast cancer. This identified superior combinations was further investigated through *in vitro* cellular assays validation and a preliminary analysis of the synergistic mechanisms. In a conclusion, this study provided a new strategy for effectively analyzing the superior synergistic combinations of anti-breast cancer compounds and understanding their complex interactions within TCM.

## 2 Materials and methods

### 2.1 Plant material, chemicals and reagents

The dried ears of *P. vulgaris* L. (PVL, Lot No. 20210116, collected from Henan province) was purchased from Xinsheng Chinese Herbal MEDICINE (Anhui, China). The whole plants of *T. mongolicum* Hand.-Mazz. (TH, Lot No. 20210420, collected from Anhui province) was purchased from Bozhou Youyuantang Pharmaceutical (Anhui, China). Scopoletin (ST) and cichoric acid (CIA) were purchased from Yuanye (Shanghai, China); caffeic acid (CA), p-coumaric acid (PCA), rosmarinic acid (ROA), esculetin (ET), rutin (RT), hypericin (HP), quercetin (QT), chlorogenic acid (CHA) and luteolin (LT) were obtained from Maclin (Shanghai, China). The purity of each compound is above 98%. Dulbecco modified Eagle medium/high glucose (DMEM) and Fetal bovine serum (FBS) were provided by Adamas Life (Shanghai, China). Cell Counting Kit-8 (CCK-8) was acquired from DOJINDO (Japan).

### 2.2 Preparation of PVL and TH extracts

PVL and TH were powdered, and ultrasonically extracted separately using 10 volumes of water or 50% ethanol (w/v) for 40 min at room temperature. The ultrasonication was conducted at a frequency of 40 kHz with a power setting of 250 W to ensure the thorough release of active components. The extracts were then filtrated, and concentrated using a rotary evaporator at 40°C under vacuum conditions, reducing the volume to a concentration of 1.0 g/mL (measured by crude drug, the same below) to eliminate the effect of ethanol on cell viability. Subsequently, the concentrated extracts were processed through centrifugation at a speed of 3,700 rpm for a duration of 10 min. The supernatants were sterilized through a 0.22 μm membrane, and then diluted with DMEM medium to create stock solutions at 100 mg/mL. Since the ratio of PVL to TH is usually recommended to be between 1:2 and 2:1 (Yan et al., 2018; Tang et al., 2020), the stock solutions of PVL and TH were mixed in volume ratios of 1:1, 1:2, and 2:1 to obtain mixed stock solutions. These were further diluted with cell culture medium to prepare a series of working solutions.

### 2.3 *In vitro* anti-tumor effects of the extracts

The human breast cancer cell line MCF-7 were obtained from the Cell Bank of the Chinese Academy of Sciences (Shanghai, China) and cultured in DMEM supplemented with 10% fetal bovine serum (FBS) in a humidified incubator at 37°C with 5% CO_2_. MCF-7 cells were seeded into 96-well plates at a density of 5 × 10^3^ cells per well. After 24 h of culture in cell culture media, the cells were treated with various groups at different concentration gradients. Each concentration was tested in triplicate. After 48 h, 10 µL of Cell Counting Kit-8 (CCK-8) solution was added to each well, and the plates were incubated at 37°C for 1 h. The absorbance was measured at 450 nm using a microplate reader to determine cell viability (Equation 1) and calculate IC_50_ values. The extract with the lowest IC_50_ value was regarded as superior extracts.

$$\text{cell vaibility }\left(\%\right)=\frac{\text{absorbance}\;\text{of}\;\text{test}\;\text{group}-\text{absorbance}\;\text{of}\;\text{blank}\;\text{group}}{\text{absorbance}\;\text{of}\;\text{control}\;\text{group}-\text{absorbance}\;\text{of}\;\text{blank}\;\text{group}}\times 100\%$$ (1)

where the test group was treated with various groups at different concentration gradients, the control group was treated with DMEM only, and the blank group, without cells and compounds, was treated with DMEM to account for background absorbance.

The combination index (CI), based on the Chou-Talalay method (Chou, 2010), was calculated using CompuSyn software to investigate the synergistic, additive, or antagonistic effects of anti-breast cancer compound combinations, where CI > 1 indicates antagonism, CI = 1 indicates additive effects, and CI < 1 indicates synergy (Equation 2).

$$\text{CI}=\frac{D_{A}}{{\text{IC}}_{50,A}}+\frac{D_{B}}{{\text{IC}}_{50,B}}$$ (2)

where D_A_ and D_B_ represent the concentration of the compounds at a 50% growth inhibition rate when used in combination, and IC_50, A_ and IC_50, B_ represent the IC_50_ values of the compound when used alone.

### 2.4 Identification of compounds in superior extracts using LC-MS analysis

PVL and TH powders were extracted with 10 volumes of 50% ethanol for 40 min, based on previous reports with modification (Psotová et al., 2003; Wu et al., 2022; Zholdasbayev et al., 2023; Liu et al., 2024). The extracts were then filtrated and concentrated to a final concentration of 1.0 g/mL. Subsequently, the extracts were diluted in ethanol at a ratio of 1:4 and airtightly refrigerated for 24 h. The mixture was filtered and further concentrated to 100 mg/mL. Following centrifugation at 12,000 rpm for 10 min, the supernatants were collected and filtered through a 0.22 μm membrane.

The Liquid Chromatograph (LC) analysis was performed on a Thermo Hypersil Gold C18 column (100 mm × 2.1 mm, 1.9 μm). The column temperature was maintained at 45°C; the injection volume was 2 μL; the flow rate was 0.35 mL/min with gradient elution. The mobile phase, consisting of acetonitrile (A) and water (B), was applied with the gradient elution as follows: 0–4 min, 2% A; 4–10 min, 2%–50% A; 10–25 min, 50%–98% A (PVL); 0–10 min, 2%–10% A; 10–18 min, 20%–50% A; 18–30 min, 50%–98% A (TH).

Mass spectrometry (MS) of the samples were conducted in negative ion mode using the HESI ion source. The data were screened and matched using Compound discoverver 3.1.0 software. The capillary voltage was set to 3,200 V, with a capillary temperature of 320°C. The sheath gas flow rate was 48.75 units, and the auxiliary gas flow rate was 11.88 unit. Full-scan mass spectra were acquired across a mass-to-charge ratio (m/z) range of 100–1,000.

### 2.5 Prediction of synergistic combinations of anti-breast cancer compounds from PVL and TH extracts

#### 2.5.1 Expanding compound-target interactions for input of DeepMDS

Due to the limitations of existing databases, we employed the compound-target correlation space-based interaction prediction model (CTCS-IPM) to explore potential interactions between compounds identified via LC-MS and 1,093 targets. These predicted interactions subsequently were then used as inputs for DeepMDS (Rui et al., 2020). Briefly, the data of 50,564 pairs of interaction consisting of 1,093 targets and 31,880 compounds were collected from the relevant database. Molecular descriptors were calculated for these compounds using Molecular Operating Environment 2020 (MOE, Chemical Computing Group ULC, Montreal, Canada), and for targets using the Protein Feature Server (ProFeat). Then, these molecular descriptors were standardized and analyzed using SPSS software through principal component analysis (PCA) calculation, resulting in 42 compound and 237 protein molecular descriptors. The principal components captured 86.64% and 89.80% of the cumulative variance, respectively. A threshold for a given compound group of each target was then established based on a 95% confidence interval of the Euclidean distance between each compound or target pair. After calculating the position of compounds and targets in the compound-protein correlation space, CTCS-IPM was able to infer potential interactions with a recall rate of 91.18%.

We also integrated known compound-target interactions from databases such as TCMSP, TCMID, and BindingDB with predictions from CTCS-IPM. This combined data formed an expanded interaction network, visualized using Cytoscape software to analyze network abundance. Subsequently, this expanded interaction network was used as input for DeepMDS to predict synergistic compound combinations.

#### 2.5.2 Prediction of synergistic combinations using DeepMDS model

To identify synergistic combinations of anti-breast cancer compound in TCM, we have developed DeepMDS model (She et al., 2022). Briefly, The DeepMDS model was constructed to predict drug synergy by integrating genomic features of cancer cell lines and drug-target interaction data. Genomic data were obtained from ArrayExpress and eBioPortal, while drug-target data were collected from GDSC, NPACT, DrugBank, PubChem, and TCMSP. These datasets were combined to generate 201,405 unique samples representing both genomic and drug-target features. The DeepMDS architecture consisted of an input layer, two dense hidden layers with 200 and 100 nodes, ReLU activation functions, and dropout layers to reduce overfitting. The output layer was designed to predict both classification outcomes (positive or negative synergy) and regression results (pseudo-IC_50_ values). The model was trained using Stochastic Gradient Descent (SGD) and the Adam optimizer, with a learning rate of 10^−5^, batch size of 128, and 200 epochs, optimizing mean square error (MSE) for regression tasks.

Therefore, this model could offer the predicted pseudo-IC_50_ values for each combination, which could be used to measure the synergistic anti-tumor effect of different combinations. In this study, before predicting compound combinations, a systematic clustering analysis was conducted on the compounds from TVL and TH utilizing the within-groups linkage method. This method helped avoid redundancy which caused by target overlap among the prediction samples. Compounds were grouped based on Euclidean distances with a threshold of 5. The selection of compounds followed two criteria: 1) priority to compounds in separate clusters to ensure diversity, and 2) in cases where compounds shared a cluster, the one with the most targets was selected.

Selected compounds were then used to construct both compound-pair and multi-compound combinations. Each combination was characterized using 215 genomic features specific to the given cancer cell line and 1,093 molecular target features of compounds, serving as individual modeling samples. The DeepMDS model was employed to predicted the synergy effects of these combinations on the MCF-7 cancer cell line. The combination with the lowest pseudo-IC_50_ value was identified as the superior synergistic anti-breast cancer combination, henceforth referred to as the superior combination.

### 2.6 Content determination of compounds contained in superior combinations in herbal extract

To measure the concentrations and ratios of the compounds in superior combinations for subsequent anti-breast cancer experiments, the contents of these compounds in the herbal extracts were determined using high-performance liquid chromatography (HPLC).

*Prunella vulgaris* L. were purchased from various regions, including Henan (Lot No. 20210116, 20210822), Hubei (Lot No. 20210906), Jiangsu (Lot No. 202101926), Sichuan (Lot No. 20220111), and Anhui (lot No. 20220103). *Taraxacum mongolicum* Hand.-Mazz. samples were obtained from Henan (Lot No. 20210814), Anhui (Lot No. 20210420, 20210925), Shanxi (Lot No. 20210510), Gansu (Lot No. 20210522), and Hunan (Lot No. 20210826). These herbal samples were authenticated by Professor Ouyang Zhen of Jiangsu University and ground into powder for experimental use. The powdered herbal sample (1.0 g) was extracted with 10 mL of either ultrapure water or 50% ethanol solution for 40 min in an ultrasonic bath. The extracts were then filtered and centrifuged at 3,700 rpm for 10 min. Subsequently, the supernatant was collected and filtered through a 0.22 µm filter membrane for analysis.

For the preparation of calibration standards, 5.0 mg each of caffeic acid, p-coumaric acid, scopoletin, rosmarinic acid, esculetin, rutin, hypericin and quercetin were dissolved in 5.0 mL of methanol to obtain a stock solution of PVL standards at a concentration of 1.0 mg/mL. Similarly, TH standards containing chlorogenic acid, cichoric acid, caffeic acid and luteolin were prepared at the same concentration of 1.0 mg/mL. These stock solutions were then diluted with methanol to produce a mixed control solution with a concentration of 100 μg/mL for each compound.

HPLC analysis was performed on an InertSustain™ ODS-C18 column (250 mm × 4.6 mm, 5 μm), with a mobile phase consisting of acetonitrile (A) and 0.1% acetic acid aqueous solution (B), flowing at a rate of 1.0 mL/min. The column temperature was maintained at 30°C, and the injection volume was set to 20 μL. The gradient elution parameters for PVL were as follows: 0–30 min, 10%–25% A; 30–50 min, 25%–45% A. Chromatograms were obtained with a detection wavelength of 325 nm for caffeic acid, p-coumaric acid, scopoletin, and rosmarinic acid, and 360 nm for esculetin, rutin, hypericin and quercetin. For TH, the gradient elution was set as 0–20 min, 10%–45% A, and 325 nm as the detection wavelength. Each compound exhibited a good linear relationship within the range of 1–100 μg/mL, and the average recoveries were 98.63%–102.36%. The relative standard deviation (RSD) values for the precision, stability, and repeatability test were all less than 3%, which met the methodological requirements.

### 2.7 *In vitro* cellular validation of the identified superior combinations

To investigate the anti-breast efficacy of the superior combinations predicted by DeepMDS, each compound in the superior combinations was initially dissolved in DMSO as stock solutions at a concentration of 400 mM. Working solutions were then prepared by diluting these stock solutions with cell culture medium, ensuring that the final DMSO concentration was less than 0.1%. To evaluate their synergistic effects, the molar ratio of compounds in superior combinations was set according to the actual content of each compound in the extracts. This setup aimed to closely simulate the natural proportions found in the herbal extracts, and to investigate whether these superior combinations could significantly contribute to the anti-tumor effects of the extracts. The test solutions for different geographical origins of PVL and TH were prepared following the method described above, using 50% ethanol as the extraction solvent.

The anti-breast cancer efficacy of these combinations was performed on human breast cancer cell line MCF-7 using CCK-8 assay. This cellular viability assay was detailed previously in Section 2.3.

### 2.8 Preliminary analysis on the anti-tumor synergistic mechanism of superior combinations

To understand the mechanisms underlying the anti-tumor effects of the superior combinations, we employed Metascape for KEGG pathway enrichment analysis on the targets of each predicted superior combination. Briefly, all targets were converted to a uniform Entrez Gene ID, and the Human species gene was selected with parameters set to Min Overlap of 3, *p*-value Cutoff of 0.01, and Min Enrichment of 1.5. KEGG Mapper was used to acquire the pathway maps based on the target enrichment for each compound in the superior combinations. Then the synergistic relationships among critical tumor pathways involved in the superior combinations were examined based on the “pathway in cancer.” Subsequently, the regulatory relationships between targets and pathways indicated in the KEGG pathway map were used to construct the “superior combination compound-target-tumor related pathway” network, which was performed by Cytoscape 3.7.2 software. Moreover, this study investigated how each compound synergistically regulates pathways to play an anti-tumor role.

### 2.9 Statistical analysis

Statistical differences between two groups were assessed using the two-tailed Student’s t-test, while differences among multiple groups were analyzed using one-way ANOVA. In all cases, *p*-value of less than 0.05 was considered to represent statistical significance.

## 3 Results

### 3.1 The anti-breast cancer effects of extracts from PVL and TH

**FIGURE 1:**
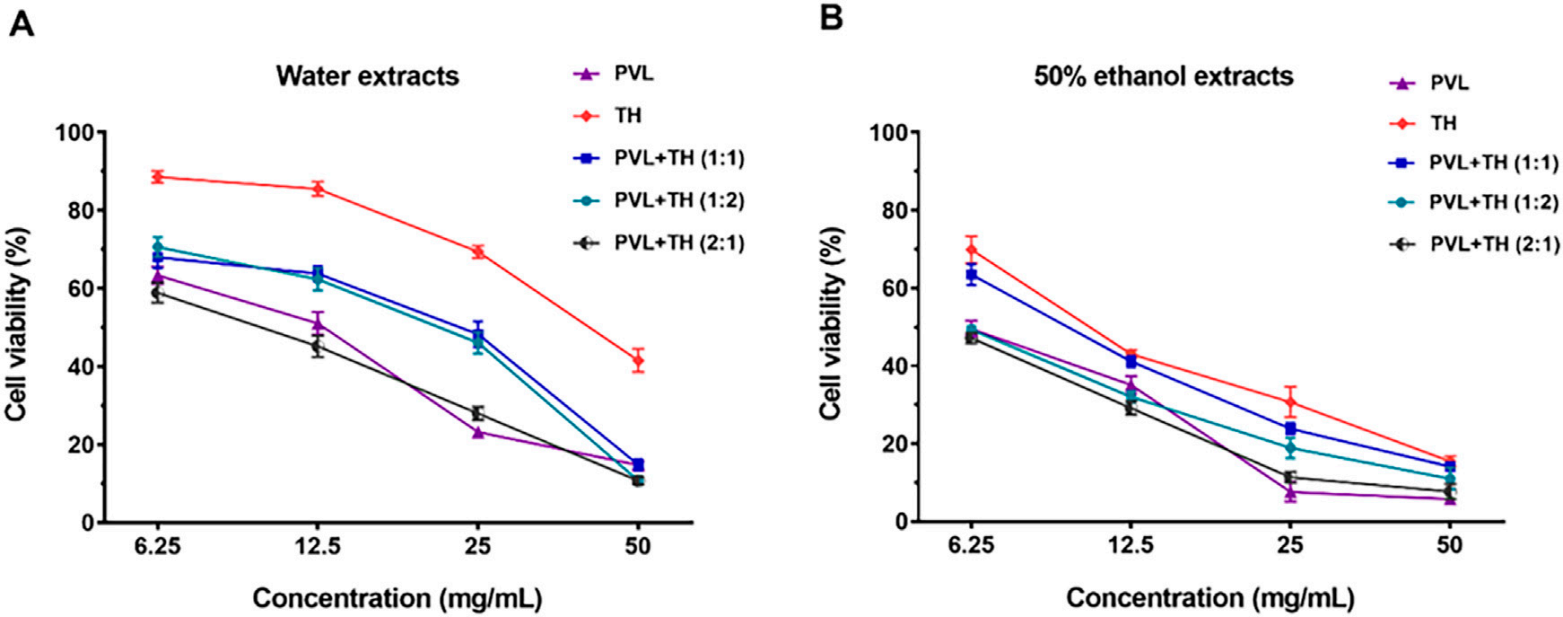
Comparative effects of *Prunella vulgaris* L. (PVL) and *Taraxacum mongolicum* Hand.-Mazz. (TH) extracts, individually and in combination, on MCF-7 cell viability. **(A)** Water extracts of PVL and TH, as well as their combinations at ratios of 1:1, 1:2, and 2:1, were evaluated for their impact on cell viability. **(B)** Effects of 50% ethanol extracts of PVL and TH, and their combinations at similar ratios, were assessed on MCF-7 cells. Data are presented as mean ± SD (n = 5).

The anti-proliferative effects of *P. vulgaris* L. (PVL) and *T. mongolicum* Hand.-Mazz. (TH) extracts on MCF-7 human breast cancer cells were investigated using a CCK-8 assay. The results demonstrated that all extracts exhibited a concentration-dependent inhibition of MCF-7 cell proliferation (Figure 1A). We also examined the cell toxicity of a 0.1% ethanol solution, and the results showed that this solution had no significant effect on the growth of cells (Supplementary Figure S1). Notably, the 50% ethanol extracts of PVL and TH displayed stronger anti-breast cancer effect compared to their water counterparts, with IC_50_ values of 6.47 mg/mL and 11.68 mg/mL, respectively. When 50% ethanol extracts of PVL and TH were combined in a 2:1 ratio, the synergistic anti-breast cancer effect was more potent, achieving an IC_50_ value of 5.54 mg/mL and a combination index (CI) of 0.73 (Figure 1B; Table 1). Therefore, the 50% ethanol extract of PVL and TH was selected as the superior extract for further investigation.

**TABLE 1:** IC_50_ and CI values of PVL and TH on MCF-7 cells alone or in combination.

Water extract	IC_50_ (mg/mL)	CI	50% ethanol extract	IC_50_ (mg/mL)	CI
PVL	10.70	—	PVL	6.47	—
TH	43.20	—	TH	11.68	—
PVL:TH (1:1)	15.91	0.93	PVL:TH (1:1)	9.61	1.15
PVL:TH (1:2)	15.06	0.70	PVL:TH (1:2)	5.96	0.65
PVL:TH (2:1)	9.57	0.67	PVL:TH (2:1)	5.54	0.73

Note: The symbol “—” implied that when PVL, or TH, was used alone, the CI, value could not be calculated.

### 3.2 Identification of compounds in superior extracts based on LC-MS

**FIGURE 2:**
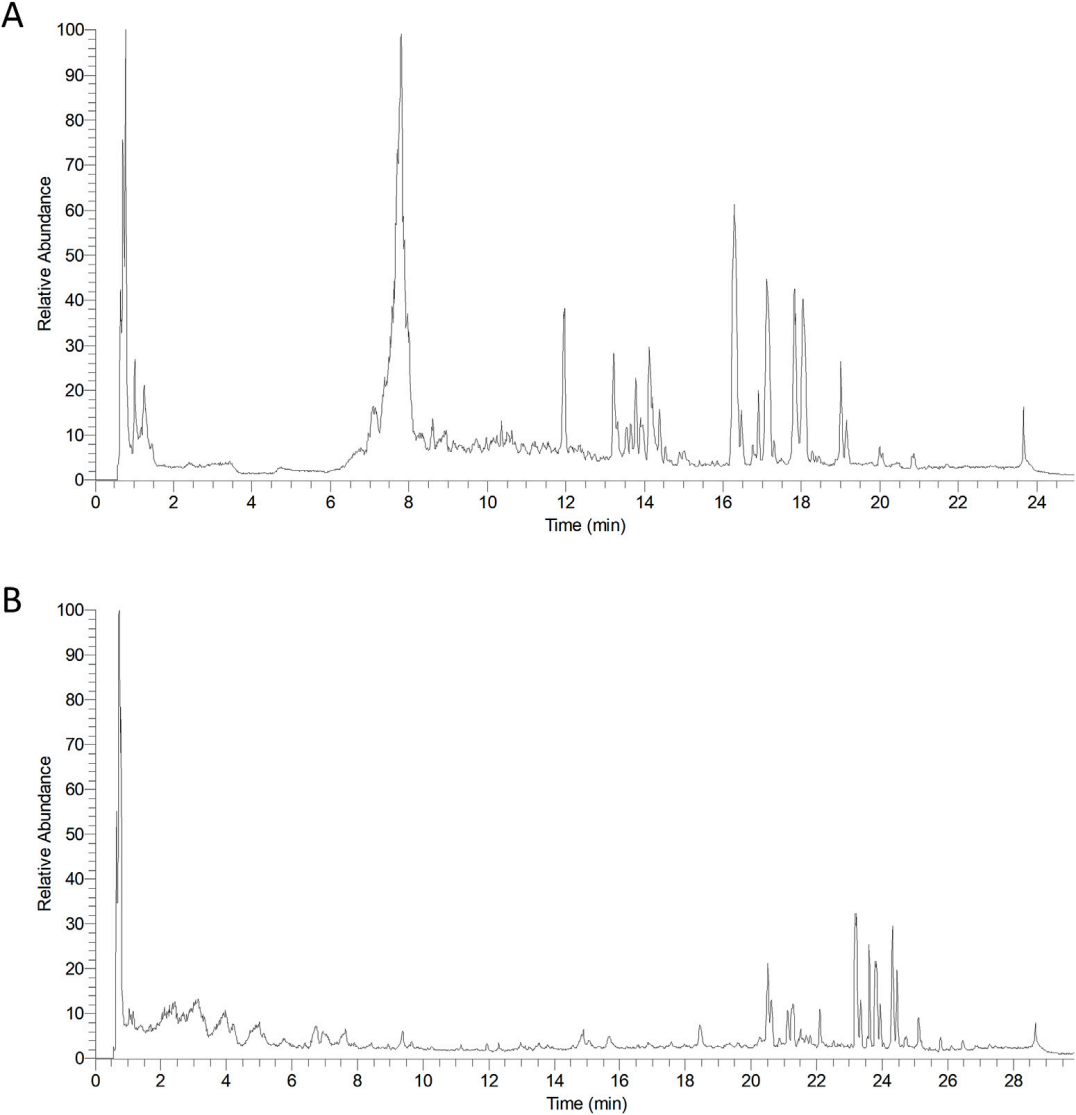
Representative total ion chromatograms of *Prunella vulgaris* L. (PVL) **(A)** and *Taraxacum mongolicum* Hand.-Mazz. (TH) **(B)** extracts. The chromatograms illustrate the chemical profiles of the two herbal extracts, and the differences in chromatographic patterns between the two extracts highlight the unique chemical compositions of PVL and TH.

The compounds from PVL and TH were retrieved using the Traditional Chinese Medicine System Pharmacology Database and Analysis Platform (TCMSP) and Traditional Chinese Medicine Integrative Database (TCMID). Data matching was conducted through Compound Discoverver 3.1.0 software. As shown in Figure 2, the total ion current chromatogram provided preliminary attributions of each compound, which were further validated through database cross-referencing and literature review.

In total, 27 compounds were identified in the PVL extract and 21 in the TH extract (Supplementary Tables S1, S2). The PVL extract was characterized predominantly by flavonoids, organic acids, coumarins and terpenoids, while the TH extract was characterized mainly by organic acids, flavonoids and terpenoids.

### 3.3 Expansion of compound-target interaction

Firstly, the known targets of the compounds identified via LC-MS were retrieved from relevant databases and literature, and subsequently intersected with 1,093 modeling characteristic targets. This data intersection yielded 628 compound-target pairs involving 24 compounds and 262 targets for PVL, and 516 pairs involving 18 compounds and 252 targets for TH.

Further, the comprehensive interactions between compounds identified in extracts and the 1,093 modeling targets were predicted using the CTCS-IPM model. The resultant expanded compound-target interaction network incorporated both known and predicted interactions, and was visualized using Cytoscape software (Supplementary Figure S2). This network demonstrated a total of 1,577 compound-target interactions involving 27 compounds and 373 targets for PVL, as well as 1,604 interactions involving 21 compounds and 377 targets for TH. These results highlighted the comprehensive nature of the expanded compounds-targets interaction network, suggesting that the compounds could work synergistically against breast cancer through multiple compounds and targets.

### 3.4 Prediction of synergistic combinations of anti-breast cancer compounds in PVL and TH extracts

**FIGURE 3:**
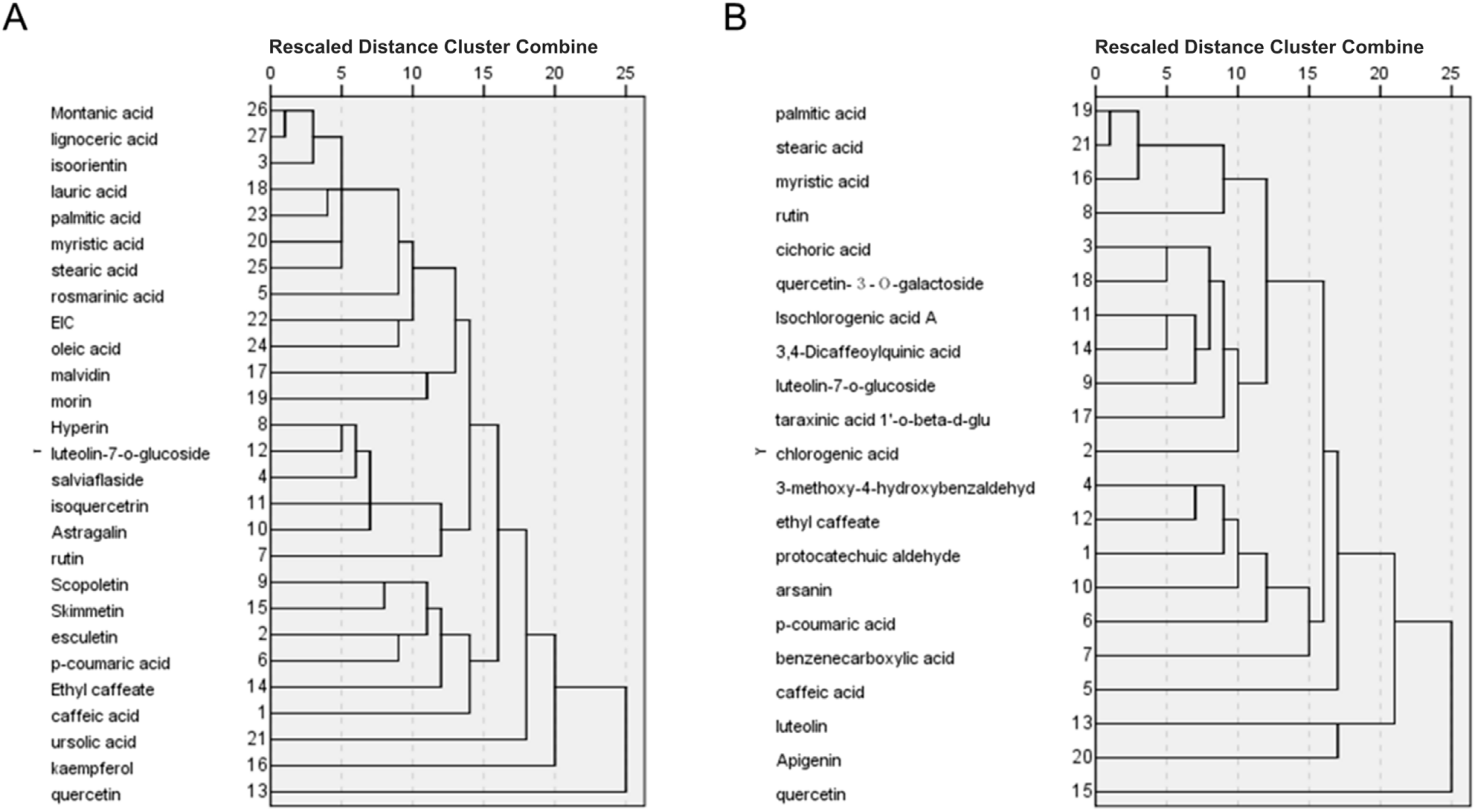
Hierarchical cluster analysis of bioactive compounds identified in PVL **(A)** and TH **(B)**. The dendrograms demonstrated the clustering of compounds based on their similarity profiles, showing the distinct chemical compositions of PVL and TH that may contribute to their observed pharmacological activities.

We adopt a within-groups linkage clustering method based on Euclidean distance to cluster the compounds. In PVL, the clustering analysis showed that montanic acid, lignoceric acid and isoorientin fell into one cluster, while lauric acid and palmitic acid fell into another. According to the number of their targets, isoorientin, lauric acid and 22 other compounds were used to construct prediction samples of PVL (Figure 3A). Similarly in TH, palmitic acid, stearic acid and myristic acid were clustered together, with stearic acid and 18 other compounds chosen for TH sample construction (Figure 3B).

For PVL, 55,454 combinations ranging from pairs to five-compound combinations were generated from 24 selected compounds, which was characterized by a feature matrix of 1,308 × 55,454 (1,308 features and 55,454 prediction samples). DeepMDS was then utilized to predict the IC_50_ values for these combinations on the MCF-7 cell line (Table 2). As a result, the combination F973, including CA, ROA, PCA, and ET, showed the lowest IC_50_ value among the 55,454 combinations. Similarly, TH yielded 16,663 combinations, with a feature matrix of 1,308 × 16,663 (1,308 features and 16,663 prediction samples). The predicted IC_50_ values of different combinations using DeepMDS were subsequently ranked, and the combination T271 (CHA, CIA, and CA) had the lowest IC_50_ value, showing its potential synergistic effect against MCF-7 cell (Table 2). Extending the analysis to the traditional combination of PVL and TH used in treating breast cancer (Yan et al., 2018), we evaluated a total of 87,222 combinations involving 36 compounds, resulting in a feature matrix of 1,308 × 87,222 (1,308 features and 8,722 prediction samples). The top candidates based on IC_50_ values were combinations T1685 (CHA, ROA, ST) and V3589 (CA, RT, LT, PCA, ROA) (Table 2). These top predicted combinations were considered as superior combinations for further investigation.

**TABLE 2:** Prediction results of different combinations from PVL, TH and PVL-TH on MCF-7 cells using DeepMDS (part).

Herb	Predicted value	Group number	Anti-breast cancer compound combinations
PVL	−1.0739	F973	Caffeic acid; Rosmarinic acid; P-coumaric acid; Esculetin
	−1.0717	V31533	P-coumaric acid; Rutin; Lauric acid; Ursolic acid; Stearic acid
	−1.0716	V1237	Caffeic acid; Esculetin; Isoquercetrin; Lauric acid; EIC
	−1.0714	V11633	Esculetin; Rosmarinic acid; Hyperin; Astragalin; Ethyl caffeate
	−1.0709	F8222	Rutin; Lauric acid; Ursolic acid; Oleic acid
	−1.0708	V22035	Isoorientin; Kaempferol; Malvidin; Lauric acid; Stearic acid
TH	−1.0999	T271	Chlorogenic acid; Cichoric acid; Caffeic acid
	−1.0902	V8914	Caffeic acid; Rutin; Ethyl caffeate; Luteolin-7-o-glu; Benzoic acid
	−1.0866	V6149	Cichoric acid; Caffeic acid; Arsanin; Ethyl caffeate; 3,4Dicaffeoylquinicacid
	−1.0836	V1794	Caffeic acid; Stearic acid; Protocatechuic aldehyde; Benzoic acid; Luteolin-7-o-glu
	−1.0835	V9118	Caffeic acid; Taraxinic acid; Stearic acid; Benzoic acid; 3,4Dicaffeoylquinicacid
	−1.0833	T111	Arsanin; Luteolin; Protocatechuic aldehyde
PVL-TH	−1.1150	T1685	Chlorogenic acid; Rosmarinic acid; Scopoletin
	−1.0961	V3859	Caffeic acid; Rutin; Luteolin; P-coumaric acid; Rosmarinic acid
	−1.0944	F2398	Caffeic acid; Oleic acid; Cichoric acid; P-coumaric acid
	−1.0941	F9448	Esculetin; Scopoletin; Lauric acid; 3,4Dicaffeoylquinicacid
	−1.0933	V29066	Scopoletin; Astragalin; Isoquercetrin; Rosmarinic acid; 3,4Dicaffeoylquinicacid
	−1.0929	F1485	Caffeic acid; Salviaflaside; EIC; Cichoric acid

Next, we expanded the combination F973, which included caffeic acid (CA), rosmarinic acid (ROA), p-coumaric acid (PCA), and esculetin (ET), by sequentially introducing rutin (RT), scopoletin (ST), quercetin (QT), and hypericin (HP). Notably, the addition of RT to F973, forming F973-1, enhanced the anti-breast cancer potential with a value of −0.4618 (Table 3). However, the addition of more compounds into F973 potentially reduced the anti-tumor effect, as higher values suggested impaired synergy.

**TABLE 3:** Prediction results of superior combination on MCF-7 cells after addition of other compounds using DeepMDS.

Predicted values	Combinations
−0.9324	T1685-2 (CHA:ROA:ST:CA:CIA)
−0.4618	F973-1 (CA:ROA:PCA:ET:RT)
−0.3310	T1685-3 (CHA:ROA:ST:CA:CIA:PCA)
−0.1312	T1685-1 (CHA:ROA:ST:CA)
−0.0530	F973-2 (CA:ROA:PCA:ET:RT:ST)
0.0112	V3859-1 (CA:RT:LT:PCA:ROA:CIA)
1.8148	V3859-3 (CA:RT:LT:PCA:ROA:CIA:CHA:ET)
1.8148	T1685-4 (CHA:ROA:ST:CA:CIA:PCA:LT)
1.8148	T1685-5 (CHA:ROA:ST:CA:CIA:PCA:LT:ET)
1.8148	T1685-6 (CHA:ROA:ST:CA:CIA:PCA:LT:ET:RT)
1.8148	F973-3 (CA:ROA:PCA:ET:RT:ST:QT)
1.8148	F973-4 (CA:ROA:PCA:ET:RT:ST:QT:HP)
1.8293	T271-1 (CHA:CIA:CA:LT)
1.8865	T271-2 (CHA:CIA:CA:LT:PCA)
1.9002	V3859-2 (CA:RT:LT:PCA:ROA:CIA:CHA)
2.3813	T271-3 (CHA:CIA:CA:LT:PCA:RT)

In contrast, extending the combination V3859 (CA, RT, luteolin (LT), PCA, and ROA) sequentially with caffeic acid isovaleryl (CIA), cichoric acid (CHA), and ET increased the value to 1.8148, indicating reduced anti-breast cancer efficacy. A similar diminishing trend was observed with T1685 (CHA, ROA, and ST), where adding six compounds (CA, CIA, PCA, LT, ET and RT) also leading to increased values, suggesting a reduction in synergistic effectiveness (Table 3).

### 3.5 Content determination of compounds in PVL and TH extracts

The contents of compounds, which involved in superior combinations, were determined using an HPLC method in extracts from PVL (Lot No. 20210116) and TH (Lot No. 20210420). As shown in Table 4, it revealed that different extraction solvents significantly affected the yield of compounds from the herbs. For example, the water extract of PVL contained 2.7 times more caffeic acid than its 50% ethanol counterpart. In contrast, the level of rosmarinic acid were three times higher in the 50% ethanol extract than in the water extract. The comparable results were observed in the TH extracts. The content of chicoric acid was greater in the aqueous extract compared to the 50% ethanol extracts, while luteolin was more abundant in the 50% ethanol extract.

**TABLE 4:** Contents of 8 compounds in PVL and TH extracts.

Compounds	Contents in PVL extracts (mg/g)		Compounds	Contents in TH extracts (mg/g)	
	Water	50% ethanol		Water	50% ethanol
Caffeic acid	1.293	0.459	Chlorogenic acid	0.517	0.422
P-coumaric acid	0.066	0.023	Cichoric acid	2.491	2.017
Scopoletin	—	0.066	Caffeic acid	0.137	0.359
Rosmarinic acid	1.564	4.397	Luteolin	0.036	0.303
Esculetin	0.039	0.036	P-coumaric acid	0.057	0.035
Rutin	0.04	0.329	Rutin	0.093	0.215
Hyperoside	—	0.182			
Quercetin	0.043	0.027			

### 3.6 *In vitro* validation of anti-breast cancer effects of superior combinations

**FIGURE 4:**
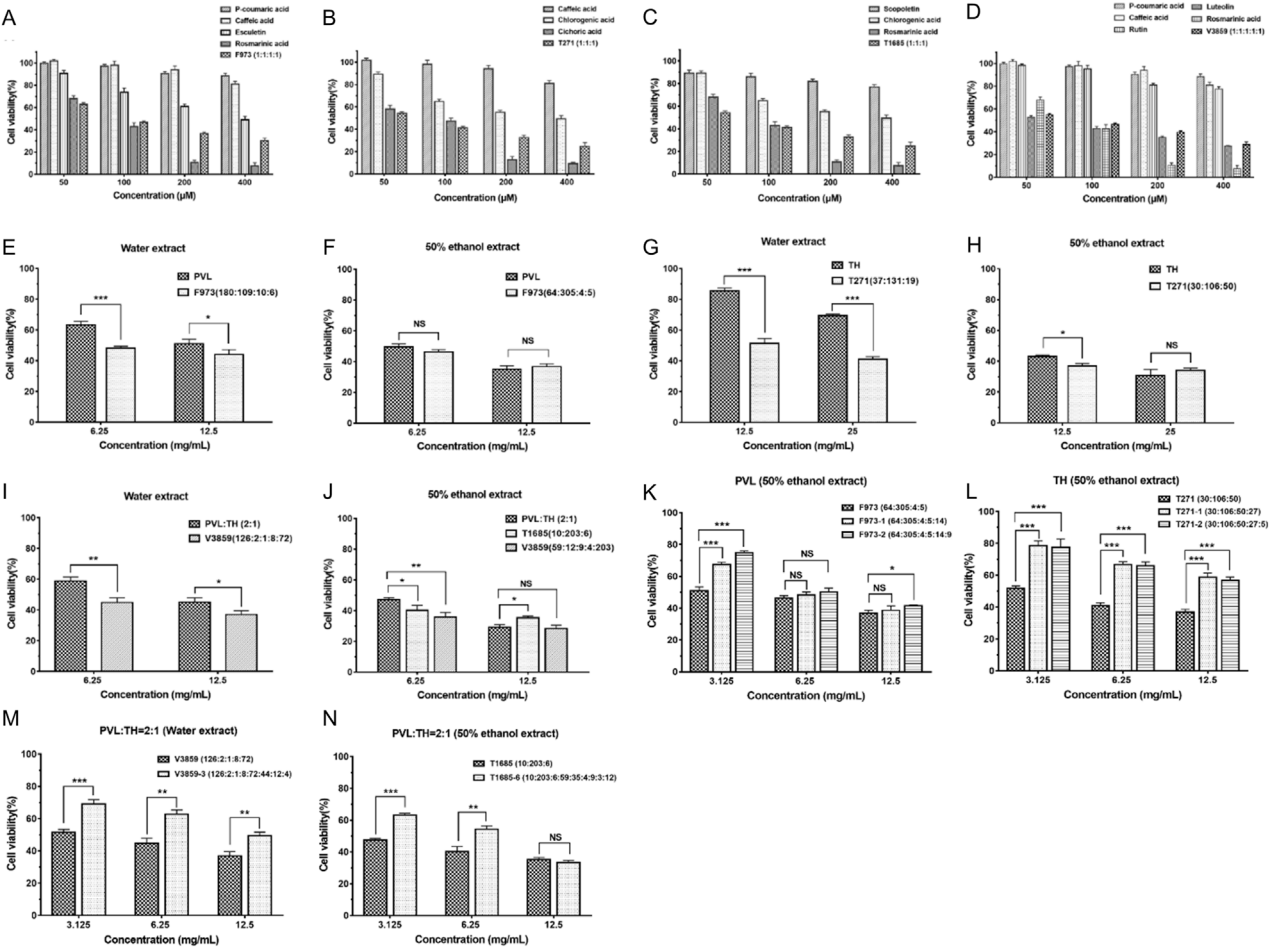
Anti-proliferative effects of individual compounds and combinations on MCF-7 breast cancer cells. **(A–D)** Effects of individual bioactive compounds and superior synergistic combinations (F973, T271, T1685, and V3859) on MCF-7 cell viability at various concentrations (50, 100, 200, and 400 μM). Each panel shows how single compounds and their superior combinations contribute to cell growth inhibition. **(E–J)** Anti-proliferative effects of superior combinations, standardized to equivalent doses of extracts, on MCF-7 cell viability. The effects of combinations, including PVL, TH, and others, are compared across different extraction methods (water and ethanol) and dosage levels (6.25, 12.5 mg/mL). **(K–N)** Evaluation of additive effects when additional compounds were included with the superior combinations, assessing MCF-7 cell viability across various concentrations. The impact of combining extracts such as PVL-TH at different ratios is also shown, providing insights into enhanced efficacy or additive benefits. *P < 0.05, **P < 0.01, ***P < 0.001; NS: not significant.

Firstly, we investigated the anti-breast cancer effects of the superior combinations in equal molar proportions and compared them with the efficacy of individual compounds under identical conditions (Figures 4A–D). In preliminary experiments of the 9 selected compounds, most were ineffective against MCF-7 cells at low concentrations (Table 5). However, rosmarinic acid, cichoric acid, and luteolin exhibited strong inhibitory effects, with IC_50_ values of 75.82, 68.86, and 59.63 μM, respectively.

**TABLE 5:** IC_50_ values of 9 single compounds on MCF-7 cells.

Compounds	IC_50_ (μM)	Compounds	IC_50_ (μM)
Scopoletin (ST)	6,607.75	P-coumaric acid (PCA)	1,132.03
Caffeic acid (CA)	885.67	Rutin (RT)	826.61
Esculetin (ET)	305.13	Chlorogenic acid (CHA)	295.18
Rosmarinic acid (ROA)	75.82	Cichoric acid (CIA)	68.86
Luteolin (LT)	59.63	—	—

The predicted superior combinations, including F973, T271, T1685 and V3859, could significantly and synergistically inhibit the proliferation of MCF-7 cells in equal molar proportions. Notably, T1685 exhibited the strongest anti-breast cancer effect, with an IC_50_ of 57.22 μM and a CI of 0.32 (Table 6). This result was in accordance with the lowest prediction value of T1685, confirming the accuracy of our DeepMDS model.

**TABLE 6:** IC_50_ and CI values of superior combination on MCF-7 under the condition of equal proportion.

Predicted value	Combination	IC_50_ (μM)	CI
−1.1150	T1685 (CHA:ROA:ST)	57.22	0.32
−1.0999	T271 (CHA:CIA:CA)	61.31	0.39
−1.0961	V3859 (CA:RT:LT:PCA:ROA)	76.46	0.51
−1.0739	F973 (CA:ROA:PCA:ET)	96.42	0.45

Next, the molar ratio of compounds in superior combinations (F973, T271, T1685, and V3859) were adjusted based on their actual content in PVL and TH extracts (Table 7). As shown in Figure 4E, combination F973 (CA:ROA:PCA:ET) demonstrated a stronger anti-proliferative effect than the water extract of PVL (6.25 mg/mL) with a ratio of 180:109:10:6 (Figure 4E), which was calculated to have an IC_50_ value of 71.86 μM and a CI value of 0.39 (Table 7). However, there was no significant difference in the anti-proliferative effect between F973 and the 50% ethanol extract at a ratio of 64:305:4:5 (Figure 4F), suggesting that the efficacy of F973 closely aligned with the composition of ethanol-based extracts. Similarly, other combinations like T271 and V3859 outperformed their corresponding extracts in anti-proliferative effects (Figures 4G–J). Notably, T1685 (CHA:ROA:ST) could only be extracted using 50% ethanol (Figure 4J). This combination showed the strongest synergistic anti-breast cancer effect with an IC_50_ value of 21.50 μM and a CI value of 0.27 (Table 7). These results indicated that these predicted superior combinations could effectively represent the anti-tumor effects of the corresponding extracts.

**TABLE 7:** Actual concentration and ratio of each superior combination in different extracts and their IC_50_ and CI values on MCF-7 cells.

Extracts (25 mg/mL)	Herbs	Combination	Ratio	Total concentration (μΜ)	IC_50_ (μM)	CI
Water extracts	PVL	F973 (CA:ROA:PCA:ET)	180:109:10:6	305	71.86	0.39
	TH	T271 (CHA:CIA:CA)	37:131:19	187	83.16	0.91
	PVL:TH (2:1)	V3859 (CA:RT:LT:PCA:ROA)	126:2:1:8:72	209	31.77	0.17
50% ethanol extracts	PVL	F973 (CA:ROA:PCA:ET)	64:305:4:5	378	59.20	0.64
	TH	T271 (CHA:CIA:CA)	30:106:50	186	21.94	0.20
	PVL:TH (2:1)	V3859 (CA:RT:LT:PCA:ROA)	59:12:9:4:203	287	23.38	0.24
	PVL:TH (2:1)	T1685 (CHA:ROA:ST)	10:203:6	219	21.50	0.27

Furthermore, we tested new combinations by incorporating additional compounds based on their content ratio in herbal extracts. Results showed that adding compounds to the original F973 and T271 generally led to reduced anti-proliferation effects (Figures 4K, L), as indicated by CI values greater than 1 (Table 8). T1685-6, despite demonstrating the strongest effect among the new combinations (Figures 4M, N), still performed weaker than the original T1685 (Table 8). The above findings were consistent with the predicted results, suggesting that an increase in the number of active compounds within a TCM combination would not necessarily translate to improved therapeutic efficacy.

**TABLE 8:** IC_50_ and CI values of superior combination on MCF-7 cells after the addition of other compounds.

Extract	Combination	IC_50_ (μM)	CI
PVL (50% ethanol)	F973-1 (64:305:4:5:14)	101.84	1.07
	F973-2 (64:305:4:5:14:9)	125.11	1.29
TH (50% ethanol)	T271-1 (30:106:50:27)	123.40	1.25
	T271-2 (30:106:50:27:5)	113.40	1.12
PVL:TH = 2:1 (Water)	V3859-3 (126:2:1:8:72:44:12:4)	121.03	0.81
PVL:TH = 2:1 (50% ethanol)	T1685-6 (10:203:6:59:35:4:9:3:12)	85.03	0.86

### 3.7 Preliminary analysis on the anti-tumor synergistic mechanisms of superior combinations

**FIGURE 5:**
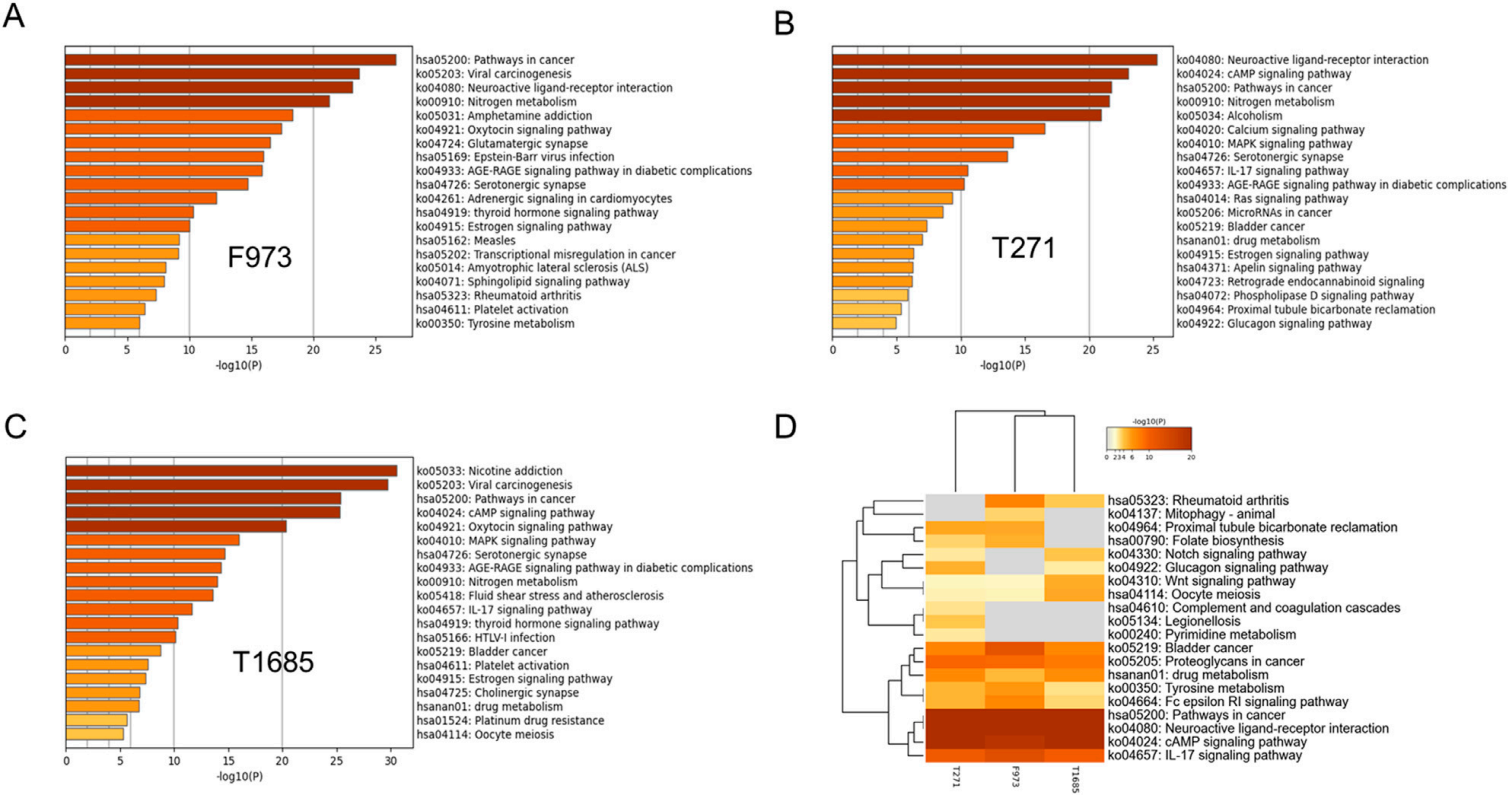
KEGG pathway enrichment analysis of superior combinations F973, T271, and T1685. **(A–C)** KEGG pathway enrichment analysis of combinations F973 **(A)**, T271 **(B)**, and T1685 **(C)**, illustrating the top pathways associated with each combination. The horizontal bars represent the -log10 (*p*-value), indicating the significance of pathway enrichment for each combination. **(D)** Comparative heatmap analysis of the enriched KEGG pathways for F973, T271, and T1685. The heatmap demonstrated the similarities and differences in pathway involvement between the combinations, providing insights into their distinct and shared mechanisms of action.

Pathway enrichment analysis was initially conducted on the targets of combination F973, T271 and T1685 using the KEGG pathway database. The top 20 enriched pathways of these combinations were compared, revealing common tumor-related pathways, including the pathway in cancer, estrogen signaling pathway, cAMP signaling pathway, and IL-17 signaling pathway (Figure 5). The combinations of T271 and T1685 were also observed to be involved in the MAPK signaling pathway and cell cycle.

Furthermore, target data of the superior combinations was mapped on hsa05200 (pathway in cancer). The enriched tumor-related pathways, along with their corresponding targets and compounds, were then visualized in a “compound-target-pathway” network using Cyoscape software.

**FIGURE 6:**
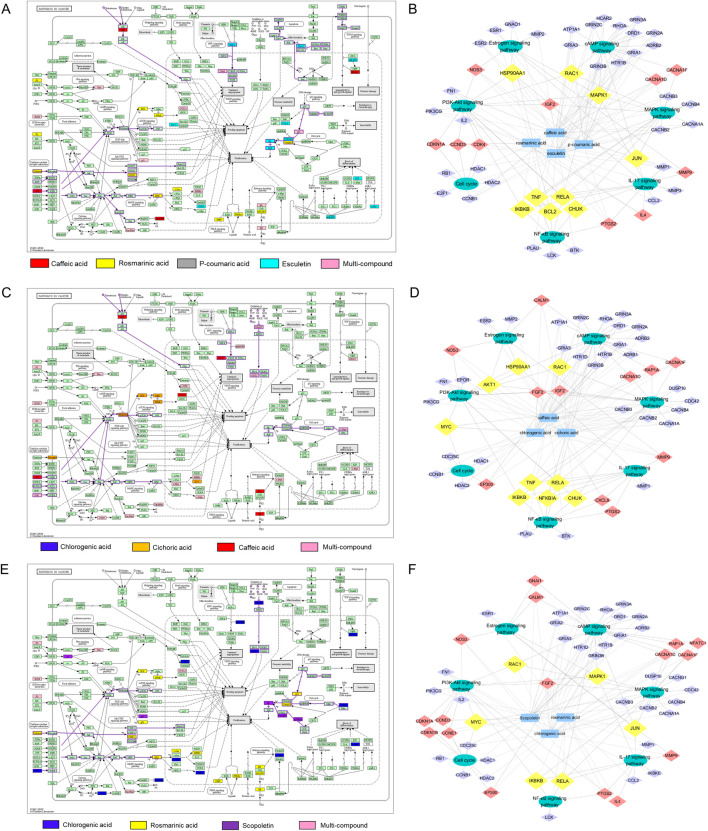
Preliminary analysis of the anti-tumor synergistic mechanisms of superior combinations F973, T271, and T1685. **(A, C, E)** KEGG pathway analysis illustrating the 'Pathways in cancer’ affected by combinations F973 **(A)**, T271 **(C)**, and T1685 **(E)**. The colored annotations indicated the specific compounds involved, including caffeic acid, rosmarinic acid, p-coumaric acid, esculetin, chlorogenic acid, and scopoletin, as well as multi-compound effects. **(B, D, F)** Synergistic compound-target-pathway networks for F973 **(B)**, T271 **(D)**, and T1685 **(F)**. These networks illustrate how the bioactive compounds interact with specific targets and the key tumor-related pathways they influence. In the network diagrams, nodes are color-coded: purple nodes represent targets present in a single pathway, red nodes indicate targets involved in two pathways, and yellow nodes signify targets associated with three or more pathways. The networks highlight the multi-target interactions contributing to the observed synergistic anti-tumor effects.

For combination F973, this network included 32 targets and multiple pathways, such as IGF-IGF1R-RAS-ERK, IGF-IGF1R-PI3K, Cytokine-Jak-STAT, p21-Cell cycle G1/S pathways (Figure 6A). The “compound-target-pathway” network diagram of F973 consists of 64 nodes (4 compound nodes, 53 target nodes and 7 pathway nodes) and 183 edges (Figure 6B). P-coumaric acid interacts with rosmarinic acid on ESR1 targets and with caffeic acid on ESR2 targets, influencing the estrogen signaling pathway. Activation of mER or GPER on the plasma membrane leads to rapid activation of other signaling pathways, including the cAMP, MAPK, and PI3K-Akt signaling pathways (Kulkoyluoglu and Madak-Erdogan, 2016). In the cAMP signaling pathway, caffeic acid, esculetin and p-coumaric acid collectively influence the downstream target RHOA, which may be associated with cell migration. Furthermore, cAMP activates the MAPK signaling pathway via the Ras-Raf-ERK pathway, sequentially impacting the cellular growth cycle, and ultimately leading to abnormal proliferation, migration and differentiation of breast cells. The PI3K-Akt signaling pathway operates concurrently with the MAPK signaling pathway, and their downstream components potentially regulate the NF-κB signaling pathway, cell cycle, apoptosis, and other related processes (Zhu et al., 2011). Additionally, F973 could also synergistically block the tumor cell cycle by down-regulating the expression of cyclins and CDKs and up-regulating the expression of the CDKs inhibitor CDKI (Zou and Lin, 2021).

In the cancer pathway map of combination T271, three compounds were involved in a total of 28 targets and multiple pathways. These pathways include IGF/FGF-GFR-RAS-ERK, IGF/FGF-GFR-PI3K, and IKK-NF-kappa B pathways (Figure 6C). The “compound-target-tumor-related pathway” network of T271 contains 61 nodes (3 compound nodes, 51 target nodes and 7 pathway nodes) and 174 edges (Figure 6D). Caffeic acid plays a crucial role in regulating the estrogen signaling pathway via ESR2, as well as other pathways involving upstream targets such as IGF2 and RAC1. In addition, chlorogenic acid and cichoric acid significantly contribute by synergistically regulating the cAMP, MAPK, and PI3K-Akt signaling pathways. These acids achieve their effects by targeting both the upstream regulator FGF2 and the downstream effector MYC. Moreover, cichoric acid could directly act on the key targets AKT1, IKBKB, CHUK, NFKBIA and RELA to regulate the NF-κB signaling pathway, which relies on IKK-mediated phosphorylation of IκBα. Cichoric acid may induce apoptosis in tumor cells by reducing NF-κB/p65 levels (Torrealba et al., 2020).

For combination T1685, the network mapped 32 targets and multiple pathways, such as FGF-FGFR-RAS-ERK, FGF-FGFR-PI3K, and MDM2-p21/p27-Cell cycle G1/S pathways (Figure 6E). The “compound-target-tumor-related pathway” network of T1685 contains 65 nodes (3 compound nodes, 55 target nodes and 7 pathway nodes) and 159 edges (Figure 6F). Chlorogenic acid regulates cAMP, MAPK, PI3K-Akt, and other signaling pathways by targeting crucial targets FGF2, RAC1, and MYC. Additionally, rosmarinic acid complements the action by targeting MAPK1, IKBKB, JUN, and RELA to synergistically inhibit the proliferation of tumor cells and induce apoptosis. In addition, the three compounds work together to regulate the cell growth cycle by targeting CDKN1B, CDKN1A, and HDAC.

### 3.8 Comparison of anti-breast cancer effects among superior extracts from different geographical origins

**FIGURE 7:**
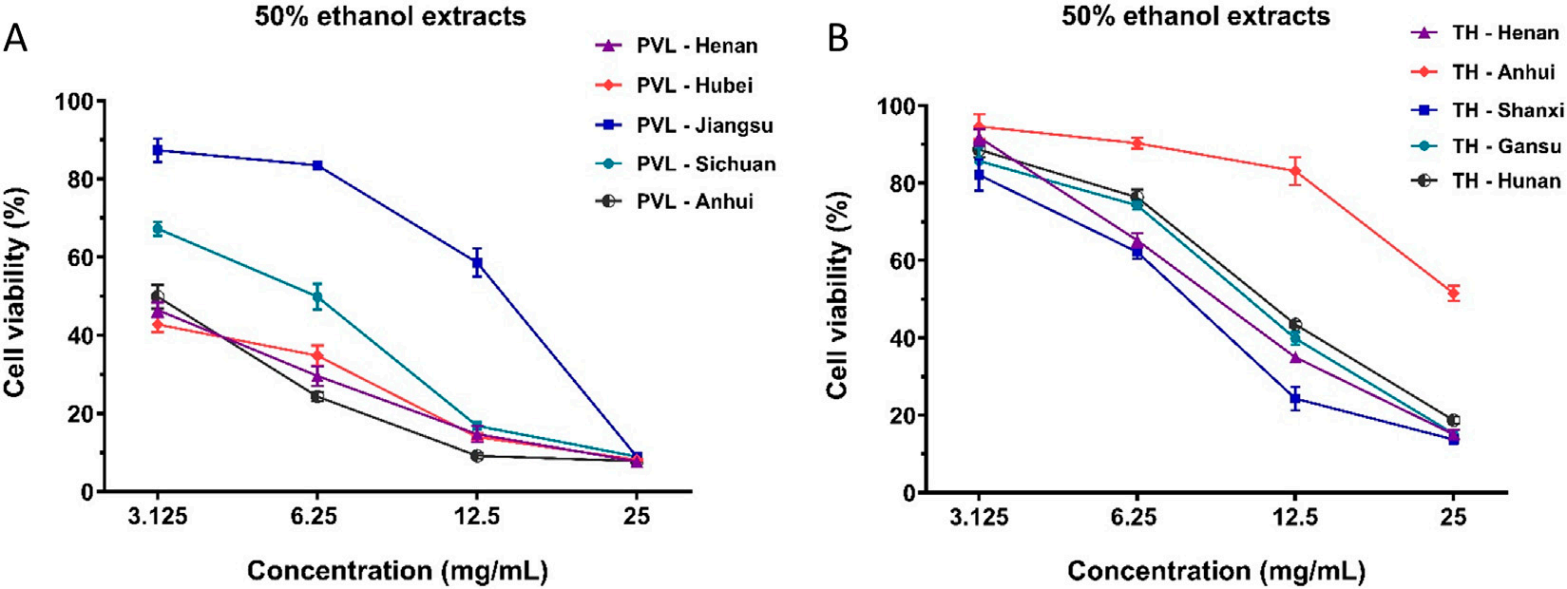
Anti-proliferative effects of PVL and TH extracts from different geographical origins on MCF-7 breast cancer cells. **(A)** Effects of PVL extracts from Henan, Hubei, Jiangsu, Sichuan, and Anhui provinces on the viability of MCF-7 cells. **(B)** Effects of TH extracts from Henan, Anhui, Shanxi, Gansu, and Hunan provinces on MCF-7 cell viability. The results demonstrated the variability in anti-proliferative efficacy of PVL and TH based on their geographical origins. Data are presented as mean ± standard deviation (n = 3).

Furthermore, we determined the content of compounds in the superior combinations of PVL and TH from different geographical origin (Supplementary Tables S3, S4). The concentration ratios and total contents of the compounds within F973 and T271 were found to correlate strongly with their anti-breast cancer effects. As shown in Figure 7A, the inhibitory effect of PVL from Anhui province (PVL-5) demonstrated the most potent anti-breast cancer effect, whereas the extract from Jiangsu province (PVL-3) was significantly less effective. This variation was attributed to the varying contents of the F973 combination within the extracts. Specifically, PVL-5, containing the highest level of F973 at 420 μM, exhibited the strongest anti-breast cancer effect. In contrast, PVL-3, with only 150 μM of F973, showed markedly reduced efficacy (Table 9). Similarly, among the 50% ethanol extracts of TH, the extract from Shanxi Province displayed the strongest anti-breast cancer effect, while the extract from Anhui Province was the least effective (Figure 7B). This was directly linked to the content of T271 in these extracts, with TH-5 (263 μM) and TH-3 (230 μM) demonstrating superior effects compared to TH-2 (136 μM), which had the lowest content of T271 (Table 9).

**TABLE 9:** Actual contents and ratios of superior combinations in various extracts from different geographical origins.

Combination	Herb extract (25 mg/mL)	Origin	Ratio	Total concentration (μΜ)
F973 (CA:ROA:PCA:ET)	PVL-1	Henan (20210822)	42:235:3:5	285
	PVL-2	Hubei (20210906)	45:269:3:4	320
	PVL-3	Jiangsu (20210926)	33:110:3:4	150
	PVL-4	Sichuan (20220111)	38:305:2:6	351
	PVL-5	Anhui (20220103)	50:364:2:5	420
T271 (CHA:CIA:CA)	TH-1	Henan (20210814)	33:120:32	186
	TH-2	Anhui (20210925)	19:90:28	136
	TH-3	Shanxi (20210510)	30:127:74	230
	TH-4	Gansu (20210522)	22:96:67	185
	TH-5	Hunan (20210826)	45:156:62	263

Beyond the total content, the specific concentration ratios of the compounds within the F973 and T271 combinations were also critical to their anti-breast cancer effects. For example, TH-2 contained higher levels of luteolin and rutin than TH-5 (Table 9). The results indicated that the efficacy of TCM extracts in cancer treatment was not only dependent on the presence of active compounds combinations but is significantly influenced by their concentration ratios and total content. This revealed the importance of considering both the quantity and ratios of multi-compound combinations in TCM for optimal anti-cancer efficacy.

## 4 Discussion

Breast cancer is a complex disease with various carcinogenic factors (Hiatt and Brody, 2018). Prolonged monotherapy in cancer treatment often results in drug resistance. Therefore synergistic compound combinations, which have been proven to reduce doses and inhibit the development of drug resistance, may be a desirable alternative in cancer treatment (Crunkhorn, 2022). TCM has been recognized as an important approach for its therapeutic potential and lower toxicity in breast cancer treatment (Gezici and Şekeroğlu, 2019; Jiang et al., 2021; Wei et al., 2023). Despite TCM’s benefits, the challenge lies in decoding its complex, multi-compound, multi-target actions to identify synergistic compound combinations and elucidate their mechanisms systematically (Hou et al., 2019; Gao et al., 2023). Moreover, the incomplete target information available in existing databases makes it difficult to gain a through grasp of the mechanisms of action of herbal compounds (Zhou et al., 2014).

Given the large number of compounds and diverse cellular targets of herbal medicines, it is impractical to discover new compound-target interactions through trial-and-error experimental methods. To address this problem, computational techniques have been employed. These include pharmacophore modeling (Wang et al., 2016), molecular docking (Guan et al., 2017), and machine learning (Olayan et al., 2018), each with its own set of challenges such as insufficient precision, computational resource constraints, and the need for extensive annotated data, respectively. In response to these challenges, we have proposed CTCS-IPM model to facilitate the prediction of interactions between multiple compounds and targets (Rui et al., 2020). By incorporating both known and predicted interactions, the CTCS-IPM model enhances the identification of synergistic compound combinations, providing a new strategy for the exploration of TCM’s potential in cancer treatment.

The traditional approach for identifying effective synergistic compound combinations proves to be both laborious and time-consuming, especially when faced with the vast number of possible compound combinations. The development of artificial intelligence, particularly deep learning, provides a potent tool for the discovery of synergistic compound combinations from extensive compound libraries (Sheng et al., 2018). However, these models primarily focus on compound pairs, leaving the potential of multi-compound combinations largely unexplored. To address this problem, we developed a deep learning-based model called DeepMDS (She et al., 2022), which integrates target information, genomic profile of cancer cell lines, and drug response data to predict synergistic anti-breast cancer compound combinations. DeepMDS emphasizes a pivotal shift towards leveraging deep learning to uncover the complex interplay of multi-compound interactions in cancer therapy.

As a classic herb pair with heat-clearing and detoxification properties, *P. vulgaris* L. (PVL)-*T. mongolicum* Hand.-Mazz. (TH) is widely applied in the clinical treatment of various types of cancer, including breast cancer (Lin et al., 2013; Deng et al., 2021; Zhang et al., 2023). PVL-TH pair is composed of several bioactive compounds that are believed to act individually, additively or synergistically to improve the anti-tumor efficacy of the herbal extracts. To identify superior combinations from PVL and TH extracts, we initially expanded the compound-target interactions to provide comprehensive target information for our synergistic compound combination prediction model, DeepMDS. Then we adopt a within-groups linkage clustering method based on Euclidean distance to cluster the compounds which identified by LC-MS to avoid redundancy caused by target duplication. Based on DeepMDS, we have identified the combinations F973 (caffeic acid, rosmarinic acid, p-coumaric acid, and esculetin), T271 (chlorogenic acid, cichoric acid, and caffeic acid), and T1685 (chlorogenic acid, rosmarinic acid, and scopoletin) as superior combinations from single PVL, single TH and PVL-TH pair, respectively. All three combinations demonstrated potent synergistic effects on MCF-7 cell proliferation, with T1685 showing the most significant effect in accordance with its predicted lowest IC_50_ value. To closely mimic the natural composition found in these extracts, we adjustments in compound ratios based on their actual content in the extracts. The results confirmed that these combinations were more effective than the extracts themselves at comparable concentrations. Therefore, the results suggested that the predicted combinations could serve as representative combinations for exerting the anti-breast cancer effects of TCM extracts.

Furthermore, we investigated how the inclusion of additional compounds affected the synergy of superior combinations predicted by DeepMDS, aiming to optimize the anti-tumor effects of these combinations. The results indicated that incorporating one or more compounds to these superior combinations did not necessarily enhance their anti-breast cancer effects, even though the added compounds themselves have demonstrated anti-breast cancer effect (such as luteolin and chlorogenic acid) (Chen et al., 2023; Wu et al., 2023). This result not only emphasized the complexity of TCM combinations, where the interplay between various compounds must be finely balanced to achieve optimal therapeutic effects, but also proved that our screening method could systematically and accurately screen out the superior synergistic anti-breast cancer compound combination.

Furthermore, the variabilities of bioactive compounds within TCM were investigated, attributed to factors such as species differences, geographical origins, and post-processing methods (Yang et al., 2005; Zou et al., 2005). By comparing the content of active compounds in different extracts, it was found that the choice of extraction solvents significantly affected the extraction rate of compounds in PVL and TH extracts. Additionally, the content of each compound and their concentration ratios also differed significantly among herbs from different geographical origins, which showed a strong correlation with the anti-breast cancer effects of herbal extracts. These results highlighted the importance of stringent regulation and standardization of medicinal materials (Liu et al., 2020). Ensuring the authenticity and quality of these materials is of great importance for maintaining the effectiveness of TCM combinations (Mahima et al., 2022).

The variability in herb combinations presents a significant challenge for standardization, affecting their consistency and therapeutic efficacy. Of particular importance is the careful optimization of content ratios within herbal combinations, as these ratios are important in determining their anti-breast cancer efficacy. While PVL and TH herbal combinations from different regions may have similar compound profiles, differences in content ratios can significantly alter their therapeutic effects. Due to the essential role of compound ratios in determining anti-tumor efficacy, the data standardization process for such herb combination needs to prioritize the detection and adjustment of these ratios. Aligning them with profiles that demonstrate optimal anti-tumor efficacy ensures that the combinations achieve their maximum therapeutic potential. This highlights the necessity of careful quantification and systematic optimization of these ratios to establish standardized and highly effective therapeutic formulations. Subsequently, standardized cultivation practices, including uniform growth conditions, can be employed to ensure consistent quality of plant materials. Controlling the geographical sourcing of raw materials and implementing rigorous post-harvest processing protocols are essential steps to reduce variability. By incorporating these standardization practices, the reproducibility, efficacy, and reliability of TCM combinations could be greatly improved, ultimately overcoming challenges posed by variability.

The study also conducted pathway enrichment analyses based on the targets of each superior combination, and further investigated their potential synergistic anti-tumor mechanism by constructing a synergistic network of “compound-target-tumor-related key pathway.” The bioinformatics analysis revealed that various compounds in superior combinations could synergistically regulate multiple tumor-related pathways by acting on multiple crucial targets, ultimately inhibiting tumor cell proliferation, inducing cell apoptosis, and blocking tumor cell cycle progression.

Combination T1685 was found to map 32 targets, involving multiple pathways, such as the FGF-FGFR-RAS-ERK, FGF-FGFR-PI3K, and MDM2-p21/p27-Cell cycle G1/S pathways. These pathways play important roles in regulating cell proliferation, survival, and cell cycle progression, and are often dysregulated in various cancers. The clinical relevance of these pathways is well established, particularly with respect to their impact on cancer progression and response to treatment. For instance, the FGF-FGFR-RAS-ERK pathway is implicated in the proliferation and differentiation of cancer cells, while the FGF-FGFR-PI3K pathway is known for its role in promoting cell survival and resistance to apoptosis. The MDM2-p21/p27-Cell cycle G1/S pathway, on the other hand, is involved in regulating cell cycle checkpoints, thereby preventing uncontrolled cell division.

Furthermore, the MAPK and PI3K-Akt pathways have been reported to be the primary downstream signaling cascades activated by the FGF-FGFR signaling pathway. These pathways have been extensively studied for their role in various cancers, including breast, lung, and colorectal cancers. Inhibitors targeting these pathways have shown promise in clinical settings. For example, the MAPK pathway, which includes key components such as RAF, MEK, and ERK, is frequently activated in cancers, leading to uncontrolled proliferation. Inhibitors such as MEK inhibitors, for example, trametinib, are used to block this pathway, effectively reducing tumor growth and improving patient outcomes in melanoma and other cancers. Similarly, the PI3K-Akt pathway plays a vital role in regulating cell survival and metabolism. Dysregulation of this pathway is associated with resistance to apoptosis and increased survival of cancer cells. PI3K inhibitors, such as alpelisib, have been approved for use in combination with endocrine therapy for hormone receptor-positive breast cancer. By mapping these pathways and identifying key molecular targets, our study provided a strong foundation for understanding how combination T1685 exerts its anti-cancer effects through modulation of these critical signaling cascades. The identification of multi-pathway interactions could reveal the potential of combination T1685 to target multiple hallmarks of cancer, offering a promising strategy for enhancing treatment efficacy and overcoming drug resistance.

To provide a more comprehensive perspective, we conceptually compared the synergistic effects of combination T1685 with those reported for established chemotherapeutic agents, such as doxorubicin combined with paclitaxel. Although no direct *in vitro* assays were performed to evaluate the efficacy of T1685 against these combinations, our analysis revealed several key distinctions. Combination T1685 demonstrated comparable efficacy in inhibiting cancer cell growth, particularly in breast cancer models. Doxorubicin and paclitaxel, when used in combination (Jin et al., 2010), have demonstrated synergistic anti-tumor effects, primarily through complementary mechanisms of action that could enhance therapeutic efficacy. However, these agents are often associated with significant toxicity, limiting their clinical application. In contrast, T1685 targets multiple signaling cascades simultaneously, including the FGF-FGFR, MAPK, and PI3K-Akt pathways. This multi-target approach not only enhances anti-cancer efficacy but also has the potential to reduce adverse effects by concurrently modulating several key characteristics of cancer. The ability to minimize toxicity while maintaining efficacy suggests that combination T1685 could serve as a promising alternative or adjunct to conventional chemotherapy, ultimately improving patient outcomes and providing a more balanced therapeutic profile.

However, one limitation of this study is that trace compounds were not completely identified in the PVL and TH extracts. These trace compounds, if identified, could potentially influence the anti-breast cancer efficacy of the extracts, especially at higher doses. Future works will be conducted to increase the raw amount for analysis to detect trace compounds within PVL and TH extracts. Incorporating these compounds into our predictive models could enhance the understanding of the extracts’ comprehensive anti-breast cancer effects and might reveal new synergistic compound combinations. In addition, there is a need for further experimental validation to elucidate the exact synergistic mechanisms of the identified superior combinations. This includes investigating how these combinations interact with specific tumor-related pathways, as well as how they affect biological processes like apoptosis and cell cycle progression. Further *in vivo* studies may provide deeper insights into the mechanisms.

## 5 Conclusion

This study employed an artificial intelligence prediction system to predict the superior synergistic combinations of anti-breast cancer compounds within TCM. The anti-tumor effects of the predicted synergistic compound combinations were comparable to those of the extracts, suggesting that these combinations could serve as the evaluation criteria for quantifying the anti-breast cancer effectiveness of TCM. Moreover, the strong correlation observed between the content of each compound and their concentration ratios among herbs from different geographical origins, and the anti-breast cancer effects of these extracts. These results shed light on the role of PVL-TH pair in breast cancer treatment and proposed a novel strategy to the quality control of TCM.

## Data Availability

The original contributions presented in the study are included in the article/Supplementary Material, further inquiries can be directed to the corresponding authors.

## References

[B1] Bansal M. Yang J. Karan C. Menden M. P. Costello J. C. Tang H. (2014). A community computational challenge to predict the activity of pairs of compounds. Nat. Biotechnol. 32 (12), 1213–1222. 10.1038/nbt.3052 25419740 PMC4399794

[B2] Baptista D. Ferreira P. G. Rocha M. (2021). Deep learning for drug response prediction in cancer. Brief. Bioinform 22 (1), 360–379. 10.1093/bib/bbz171 31950132

[B3] Chen C. Liu Y. Shen Y. Zhu L. Yao L. Wang X. (2023). Rosmarinic acid, the active component of Rubi Fructus, induces apoptosis of SGC-7901 and HepG2 cells through mitochondrial pathway and exerts anti-tumor effect. Naunyn Schmiedeb. Arch. Pharmacol. 396 (12), 3743–3755. 10.1007/s00210-023-02552-z PMC10643355 37338574

[B4] Chen G. Tsoi A. Xu H. Zheng W. J. (2018). Predict effective drug combination by deep belief network and ontology fingerprints. J. Biomed. Inf. 85, 149–154. 10.1016/j.jbi.2018.07.024 30081101

[B5] Chen Y. Zhang X. Guo Q. Cao L. Qin Q. Li C. (2019). Plant morphology, physiological characteristics, accumulation of secondary metabolites and antioxidant activities of Prunella vulgaris L. under UV solar exclusion. Biol. Res. 52 (1), 17. 10.1186/s40659-019-0225-8 30935421 PMC6442409

[B6] Chou T. C. (2010). Drug combination studies and their synergy quantification using the Chou-Talalay method. Cancer Res. 70 (2), 440–446. 10.1158/0008-5472.Can-09-1947 20068163

[B7] Crunkhorn S. (2022). Identifying synergistic drug combinations. Nat. Rev. Drug Discov. 21 (4), 260. 10.1038/d41573-022-00041-1 35256796

[B8] Deng X. X. Jiao Y. N. Hao H. F. Xue D. Bai C. C. Han S. Y. (2021). Taraxacum mongolicum extract inhibited malignant phenotype of triple-negative breast cancer cells in tumor-associated macrophages microenvironment through suppressing IL-10/STAT3/PD-L1 signaling pathways. J. Ethnopharmacol. 274, 113978. 10.1016/j.jep.2021.113978 33716082

[B9] Fan K. Cheng L. Li L. (2021). Artificial intelligence and machine learning methods in predicting anti-cancer drug combination effects. Brief. Bioinform 22 (6), bbab271. 10.1093/bib/bbab271 34347041 PMC8574962

[B10] Gao G. Ge R. Li Y. Liu S. (2019). Luteolin exhibits anti-breast cancer property through up-regulating miR-203. Artif. Cells Nanomed Biotechnol. 47 (1), 3265–3271. 10.1080/21691401.2019.1646749 31368817

[B11] Gao S. Tan H. Gang J. (2024). Inhibition of hepatocellular carcinoma cell proliferation through regulation of the Cell Cycle, AGE-RAGE, and Leptin signaling pathways by a compound formulation comprised of andrographolide, wogonin, and oroxylin A derived from Andrographis Paniculata(Burm.f.) Nees. J. Ethnopharmacol. 329, 118001. 10.1016/j.jep.2024.118001 38467318

[B12] Gao S. Tan H. Li D. (2023). Oridonin suppresses gastric cancer SGC-7901 cell proliferation by targeting the TNF-alpha/androgen receptor/TGF-beta signalling pathway axis. J. Cell Mol. Med. 27 (18), 2661–2674. 10.1111/jcmm.17841 37431884 PMC10494293

[B13] Gao W. Xu H. (2019). Root extract of Prunella vulgaris inhibits *in vitro* and *in vivo* carcinogenesis in MCF-5 human breast carcinoma via suppression of angiogenesis, induction of apoptosis, cell cycle arrest and modulation of PI3K/AKT signalling pathway. J. buon 24 (2), 549–554. 31128004

[B14] Ge B. J. Zhao P. Li H. T. Sang R. Wang M. Zhou H. Y. (2021). Taraxacum mongolicum protects against Staphylococcus aureus-infected mastitis by exerting anti-inflammatory role via TLR2-NF-κB/MAPKs pathways in mice. J. Ethnopharmacol. 268, 113595. 10.1016/j.jep.2020.113595 33212175

[B15] Gezici S. Şekeroğlu N. (2019). Current perspectives in the application of medicinal plants against cancer: novel therapeutic agents. Anticancer Agents Med. Chem. 19 (1), 101–111. 10.2174/1871520619666181224121004 30582485

[B16] Guan H. W. Xu L. J. Dong H. (2017). Application of reverse molecular docking technology in target prediction, active ingredient screening and action mechanism exploration of traditional Chinese medicine. Zhongguo Zhong Yao Za Zhi 42 (23), 4537–4541. 10.19540/j.cnki.cjcmm.20170928.021 29376249

[B17] Guo Q. Qu H. Zhang H. Zhong X. (2021). Prunella vulgaris L. Attenuates experimental autoimmune thyroiditis by inhibiting HMGB1/TLR9 signaling. Drug Des. Devel Ther. 15, 4559–4574. 10.2147/dddt.S325814 PMC8576104 34764638

[B18] Hiatt R. A. Brody J. G. (2018). Environmental determinants of breast cancer. Annu. Rev. Public Health 39, 113–133. 10.1146/annurev-publhealth-040617-014101 29328875

[B19] Hong R. Xu B. (2022). Breast cancer: an up-to-date review and future perspectives. Cancer Commun. (Lond) 42, 913–936. 10.1002/cac2.12358 36074908 PMC9558690

[B20] Hou J.-J. Zhang J.-Q. Yao C.-L. Bauer R. Khan I. A. Wu W.-Y. (2019). Deeper chemical perceptions for better traditional Chinese medicine standards. Engineering 5 (1), 83–97. 10.1016/j.eng.2018.12.005

[B21] Jaaks P. Coker E. A. Vis D. J. Edwards O. Carpenter E. F. Leto S. M. (2022). Effective drug combinations in breast, colon and pancreatic cancer cells. Nature 603 (7899), 166–173. 10.1038/s41586-022-04437-2 35197630 PMC8891012

[B22] Jiang H. Li M. Du K. Ma C. Cheng Y. Wang S. (2021). Traditional Chinese medicine for adjuvant treatment of breast cancer: taohong siwu decoction. Chin. Med. 16 (1), 129. 10.1186/s13020-021-00539-7 34857023 PMC8638166

[B23] Jin C. Li H. He Y. He M. Bai L. Cao Y. (2010). Combination chemotherapy of doxorubicin and paclitaxel for hepatocellular carcinoma *in vitro* and *in vivo* . J. Cancer Res. Clin. Oncol. 136 (2), 267–274. 10.1007/s00432-009-0658-5 19693537 PMC11827829

[B24] Jin L. Chun J. Pan C. Li D. Lin R. Alesi G. N. (2018). MAST1 drives cisplatin resistance in human cancers by rewiring cRaf-independent MEK activation. Cancer Cell 34 (2), 315–330. 10.1016/j.ccell.2018.06.012 30033091 PMC6092215

[B25] Kang L. Miao M. S. Song Y. G. Fang X. Y. Zhang J. Zhang Y. N. (2021). Total flavonoids of Taraxacum mongolicum inhibit non-small cell lung cancer by regulating immune function. J. Ethnopharmacol. 281, 114514. 10.1016/j.jep.2021.114514 34384846

[B26] Kim S. H. Huang C. Y. Tsai C. Y. Lu S. Y. Chiu C. C. Fang K. (2012). The aqueous extract of Prunella vulgaris suppresses cell invasion and migration in human liver cancer cells by attenuating matrix metalloproteinases. Am. J. Chin. Med. 40 (3), 643–656. 10.1142/s0192415x12500486 22745076

[B27] Kuenzi B. M. Park J. Fong S. H. Sanchez K. S. Lee J. Kreisberg J. F. (2020). Predicting drug response and synergy using a deep learning model of human cancer cells. Cancer Cell 38 (5), 672–684. 10.1016/j.ccell.2020.09.014 33096023 PMC7737474

[B28] Kulkoyluoglu E. Madak-Erdogan Z. (2016). Nuclear and extranuclear-initiated estrogen receptor signaling crosstalk and endocrine resistance in breast cancer. Steroids 114, 41–47. 10.1016/j.steroids.2016.06.007 27394959

[B29] Kuru H. I. Tastan O. Cicek A. E. (2022). MatchMaker: a deep learning framework for drug synergy prediction. IEEE/ACM Trans. Comput. Biol. Bioinform 19 (4), 2334–2344. 10.1109/tcbb.2021.3086702 34086576

[B30] Lin W. Zheng L. Zhuang Q. Zhao J. Cao Z. Zeng J. (2013). Spica prunellae promotes cancer cell apoptosis, inhibits cell proliferation and tumor angiogenesis in a mouse model of colorectal cancer via suppression of stat3 pathway. BMC Complement. Altern. Med. 13, 144. 10.1186/1472-6882-13-144 23800091 PMC3729539

[B31] Liu X. Zhang Y. Wu M. Ma Z. Huang Z. Tian F. (2020). The scientific elucidation of daodi medicinal materials. Chin. Med. 15, 86. 10.1186/s13020-020-00367-1 32843892 PMC7439724

[B32] Liu Y. Shi Y. Zou J. Zhang X. Zhai B. Guo D. (2024). Extraction, purification, structural features, biological activities, modifications, and applications from Taraxacum mongolicum polysaccharides: a review. Int. J. Biol. Macromol. 129193. 10.1016/j.ijbiomac.2023.129193 38191106

[B33] Luo H. Zhao L. Li Y. Xia B. Lin Y. Xie J. (2022). An *in vivo* and *in vitro* assessment of the anti-breast cancer activity of crude extract and fractions from Prunella vulgaris L. Heliyon 8 (11), e11183. 10.1016/j.heliyon.2022.e11183 36345524 PMC9636486

[B34] MacGowan A. P. Holt H. A. Reeves D. S. (1990). *In-vitro* synergy testing of nine antimicrobial combinations against Listeria monocytogenes. J. Antimicrob. Chemother. 25 (4), 561–566. 10.1093/jac/25.4.561 2112538

[B35] Mahima K. Sunil Kumar K. N. Rakhesh K. V. Rajeswaran P. S. Sharma A. Sathishkumar R. (2022). Advancements and future prospective of DNA barcodes in the herbal drug industry. Front. Pharmacol. 13, 947512. 10.3389/fphar.2022.947512 36339543 PMC9635000

[B36] Mahmoud M. A. Okda T. M. Omran G. A. Abd-Alhaseeb M. M. (2021). Rosmarinic acid suppresses inflammation, angiogenesis, and improves paclitaxel induced apoptosis in a breast cancer model via NF3 κB-p53-caspase-3 pathways modulation. J. Appl. Biomed. 19 (4), 202–209. 10.32725/jab.2021.024 34907739

[B37] National Pharmacopoeia Commission (2020). “Pharmacopoeia of the people's Republic of China: volume one,”, 292-293. Beijing: China Medical Science Press, 367.

[B38] Olayan R. S. Ashoor H. Bajic V. B. (2018). DDR: efficient computational method to predict drug-target interactions using graph mining and machine learning approaches. Bioinformatics 34 (7), 1164–1173. 10.1093/bioinformatics/btx731 29186331 PMC5998943

[B39] Pace L. E. Shulman L. N. (2016). Breast cancer in sub-saharan africa: challenges and opportunities to reduce mortality. Oncologist 21 (6), 739–744. 10.1634/theoncologist.2015-0429 27091419 PMC4912363

[B40] Preuer K. Lewis R. P. I. Hochreiter S. Bender A. Bulusu K. C. Klambauer G. (2018). DeepSynergy: predicting anti-cancer drug synergy with Deep Learning. Bioinformatics 34 (9), 1538–1546. 10.1093/bioinformatics/btx806 29253077 PMC5925774

[B41] Psotová J. Kolář M. Soušek J. Švagera Z. Vičar J. Ulrichová J. (2003). Biological activities of Prunella vulgaris extract. Phytotherapy Res. An Int. J. Devoted Pharmacol. Toxicol. Eval. Nat. Prod. Deriv. 17 (9), 1082–1087. 10.1002/ptr.1324 14595592

[B42] Rui M. Pang H. Ji W. Wang S. Yu X. Wang L. (2020). Development of simultaneous interaction prediction approach (SiPA) for the expansion of interaction network of traditional Chinese medicine. Chin. Med. 15, 90. 10.1186/s13020-020-00369-z 32863859 PMC7448979

[B43] She S. Chen H. Ji W. Sun M. Cheng J. Rui M. (2022). Deep learning-based multi-drug synergy prediction model for individually tailored anti-cancer therapies. Front. Pharmacol. 13, 1032875. 10.3389/fphar.2022.1032875 36588694 PMC9797718

[B44] Sheng Z. Sun Y. Yin Z. Tang K. Cao Z. (2018). Advances in computational approaches in identifying synergistic drug combinations. Brief. Bioinform 19 (6), 1172–1182. 10.1093/bib/bbx047 28475767

[B45] Sopirala M. M. Mangino J. E. Gebreyes W. A. Biller B. Bannerman T. Balada-Llasat J. M. (2010). Synergy testing by Etest, microdilution checkerboard, and time-kill methods for pan-drug-resistant Acinetobacter baumannii. Antimicrob. Agents Chemother. 54 (11), 4678–4683. 10.1128/aac.00497-10 20713678 PMC2976112

[B46] Tang J. W. Zheng S. Cao X. S. Zhang B. Y. Gu H. Guo Y. J. (2020). Research on the mechanism of“Dandelion-selfheal”in treatment of breast cancer based on network Pharmacology. Eval. Anal. drug-use Hosp. 20 (01), 44–49. 10.14009/j.issn.1672-2124.2020.01.011

[B47] Torrealba N. Vera R. Fraile B. Martínez-Onsurbe P. Paniagua R. Royuela M. (2020). TGF-β/PI3K/AKT/mTOR/NF-kB pathway. Clinicopathological features in prostate cancer. Aging Male 23 (5), 801–811. 10.1080/13685538.2019.1597840 30973040

[B48] Wang M. Xie D. Zhang M. Wang X.-J. (2023). Multiple ingredients of a Chinese medicine formula Sheng-Mai-San coordinately attenuate doxorubicin-induced cardiotoxicity. Pharmacol. Res. - Mod. Chin. Med. 8, 100281. 10.1016/j.prmcm.2023.100281

[B49] Wang S. Hao H. F. Jiao Y. N. Fu J. L. Guo Z. W. Guo Y. (2022). Dandelion extract inhibits triple-negative breast cancer cell proliferation by interfering with glycerophospholipids and unsaturated fatty acids metabolism. Front. Pharmacol. 13, 942996. 10.3389/fphar.2022.942996 36147318 PMC9486077

[B50] Wang X. Pan C. Gong J. Liu X. Li H. (2016). Enhancing the enrichment of pharmacophore-based target prediction for the polypharmacological profiles of drugs. J. Chem. Inf. Model 56 (6), 1175–1183. 10.1021/acs.jcim.5b00690 27187084

[B51] Wei Z. Chen J. Zuo F. Guo J. Sun X. Liu D. (2023). Traditional Chinese Medicine has great potential as candidate drugs for lung cancer: a review. J. Ethnopharmacol. 300, 115748. 10.1016/j.jep.2022.115748 36162545

[B52] Wu D.-T. Li F. Feng K.-L. Hu Y.-C. Gan R.-Y. Zou L. (2022). A comparison on the physicochemical characteristics and biological functions of polysaccharides extracted from Taraxacum mongolicum by different extraction technologies. J. Food Meas. Charact. 16 (4), 3182–3195. 10.1007/s11694-022-01439-6

[B53] Wu L. Lin Y. Gao S. Wang Y. Pan H. Wang Z. (2023). Luteolin inhibits triple-negative breast cancer by inducing apoptosis and autophagy through SGK1-FOXO3a-BNIP3 signaling. Front. Pharmacol. 14, 1200843. 10.3389/fphar.2023.1200843 37346292 PMC10279868

[B54] Yan Q. Zhang K. Li Q. Xia B. (2018). Screening of the appropriate proportion on anti-breast cancer activity of prunellae spica combined with taraxaci herba and its anti-tumor research *in vivo* . Chin. Pharm. J. 53, 10. 10.11669/cpj.2018.10.004

[B55] Yang L. W. Wu D. H. Tang X. Peng W. Wang X. R. Ma Y. (2005). Fingerprint quality control of Tianjihuang by high-performance liquid chromatography-photodiode array detection. J. Chromatogr. A 1070 (1-2), 35–42. 10.1016/j.chroma.2005.02.081 15861785

[B56] Yu Q. Xu C. Song J. Jin Y. Gao X. (2024). Mechanisms of traditional Chinese medicine/natural medicine in HR-positive breast cancer: a comprehensive literature review. J. Ethnopharmacol. 319 (Pt 3), 117322. 10.1016/j.jep.2023.117322 37866466

[B57] Zhang Q. Chen X. Palen K. Johnson B. Bui D. Xiong D. (2023). Cancer chemoprevention with PV-1, a novel Prunella vulgaris-containing herbal mixture that remodels the tumor immune microenvironment in mice. Front. Immunol. 14, 1196434. 10.3389/fimmu.2023.1196434 38077406 PMC10704350

[B58] Zhang T. Zhang L. Payne P. R. O. Li F. (2021). Synergistic drug combination prediction by integrating multiomics data in deep learning models. Methods Mol. Biol. 2194, 223–238. 10.1007/978-1-0716-0849-4_12 32926369

[B59] Zholdasbayev M. E. Atazhanova G. A. Musozoda S. Poleszak E. (2023). Prunella vulgaris L.: an updated overview of botany, chemical composition, extraction methods, and biological activities. Pharmaceuticals 16 (8), 1106. 10.3390/ph16081106 37631021 PMC10460042

[B60] Zhou H. Zhang M. Cao H. Du X. Zhang X. Wang J. (2023). Research progress on the synergistic anti-tumor effect of natural anti-tumor components of Chinese herbal medicine combined with chemotherapy drugs. Pharm. (Basel) 16 (12), 1734. 10.3390/ph16121734 PMC10748242 38139860

[B61] Zhou J. Zhou T. Jiang M. Wang X. Liu Q. Zhan Z. (2014). Research progress on synergistic anti-tumor mechanisms of compounds in traditional Chinese medicine. J. Tradit. Chin. Med. 34 (1), 100–105. 10.1016/s0254-6272(14)60062-5 25102699

[B62] Zhu C. Qi X. Chen Y. Sun B. Dai Y. Gu Y. (2011). PI3K/Akt and MAPK/ERK1/2 signaling pathways are involved in IGF-1-induced VEGF-C upregulation in breast cancer. J. Cancer Res. Clin. Oncol. 137 (11), 1587–1594. 10.1007/s00432-011-1049-2 21904903 PMC11828124

[B63] Zhu H. Zhao H. Zhang L. Xu J. Zhu C. Zhao H. (2017). Dandelion root extract suppressed gastric cancer cells proliferation and migration through targeting lncRNA-CCAT1. Biomed. Pharmacother. 93, 1010–1017. 10.1016/j.biopha.2017.07.007 28724210

[B64] Zou P. Hong Y. Koh H. L. (2005). Chemical fingerprinting of Isatis indigotica root by RP-HPLC and hierarchical clustering analysis. J. Pharm. Biomed. Anal. 38 (3), 514–520. 10.1016/j.jpba.2005.01.022 15925253

[B65] Zou T. Lin Z. (2021). The involvement of ubiquitination machinery in cell cycle regulation and cancer progression. Int. J. Mol. Sci. 22 (11), 5754. 10.3390/ijms22115754 34072267 PMC8198665

