# Supplementary material for: Artificial intelligence-driven identification and mechanistic exploration of synergistic anti-breast cancer compound combinations from Prunella vulgaris L.-Taraxacum mongolicum Hand.-Mazz. herb pair
Source: Front Pharmacol. 2025 Jan 7;15:1522787. doi: 10.3389/fphar.2024.1522787 (PMC11747269; doi:10.3389/fphar.2024.1522787)
Supplement: Supplementary file 1 [file DataSheet1.docx]

Supplementary Material

**Artificial intelligence-driven identification and mechanistic exploration of synergistic anti-breast cancer compound combinations from *Prunella vulgaris* L.-*Taraxacum mongolicum* Hand.-Mazz. herb pair**

**Chunlai Feng^a^*, Jiaxi Cheng^a^, Mengqiu Sun^a^, Chunxue Qiao^a^, Qiuqi Feng^a^, Naying Fang^a^, Yingying Ge^a^, Mengjie Rui^a^***

^a^School of Pharmacy, Jiangsu University, Zhenjiang 212013, China

**Table of Contents**

**Supplemental Figures and Figure Legends**

[Supplementary Figure 1 Effects of 0.1% ethanol on the viability of MCF-7 cells. 1](#_Toc181638539)

[Supplementary Figure 2 Expanded compound-target interaction network of (A) PVL and (B) TH. 1](#_Toc181638540)

**Supplemental Tables**

Table S1 Identification results of chemical compounds in the PVL sample 2

Table S2 Identification results of chemical compounds in the TH sample 3

Table S3 Content determination results of 8 compounds in PVL from different origins 5

Table S4 Content determination results of 6 compounds in TH from different origins 6

# Supplementary Figures and Tables

## Supplementary Figures


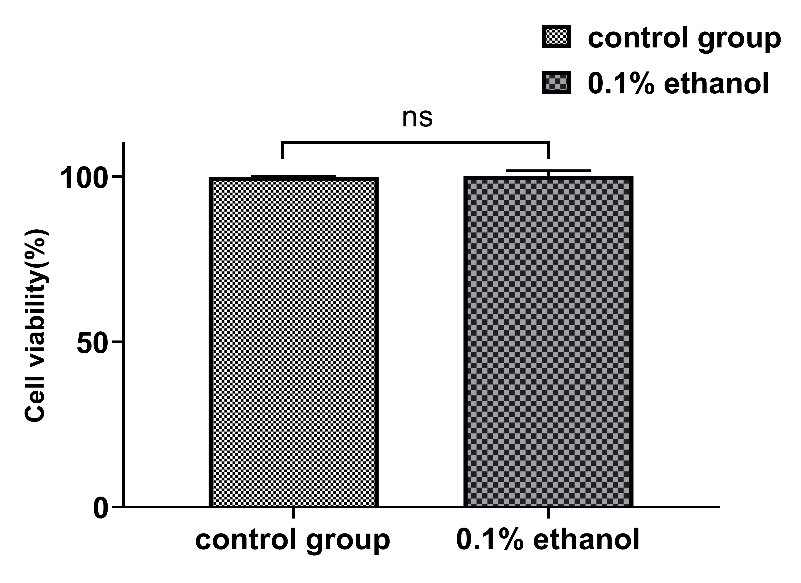


Supplementary Figure 1 Effects of 0.1% ethanol on the viability of MCF-7 cells.


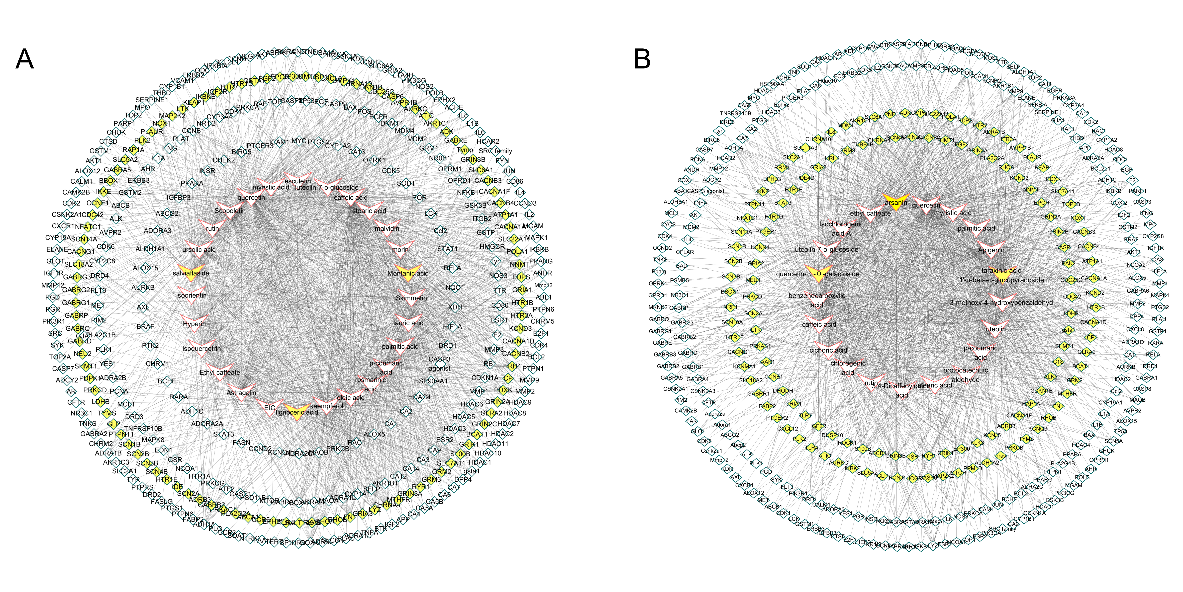


Supplementary Figure 2 Expanded compound-target interaction network of (A) PVL and (B) TH.

The pink nodes stand for compounds, the green nodes for targets, the yellow nodes for predicted additional compounds and their potential targets, and the edges exhibit the interactions between the compounds and the targets。

## Supplementary Tables

Table S1 Identification results of chemical compounds in the PVL sample

| Num | t_R_/min | Identified results | Formula | *m/z*  theoretical | *m/z*  measured | Error/  ppm | Detection mode |
| --- | --- | --- | --- | --- | --- | --- | --- |
| 1 | 2.11 | caffeic acid | C_9_H_8_O_4_ | 179.0350 | 179.0344 | 3.2508 | [M-H]- |
| 2 | 2.19 | p-coumaric acid | C_9_H_8_O_3_ | 163.0401 | 163.0392 | 5.3202 | [M-H]- |
| 3 | 2.54 | scopoletin | C_10_H_8_O_4_ | 191.0350 | 191.0345 | 2.5231 | [M-H]- |
| 4 | 7.11 | salviaflaside | C_24_H_26_O_13_ | 521.1301 | 521.1306 | 1.0287 | [M-H]- |
| 5 | 7.72 | rosmarinic acid | C_18_H_16_O_8_ | 359.0772 | 359.0778 | 1.5576 | [M-H]- |
| 6 | 7.74 | skimmetin | C_9_H_6_O_3_ | 161.0244 | 161.0235 | 5.6973 | [M-H]- |
| 7 | 8.12 | isoquercetrin | C_21_H_20_O_12_ | 463.0882 | 463.0864 | 3.8852 | [M-H]- |
| 8 | 8.19 | rutin | C_27_H_30_O_16_ | 609.1461 | 609.1466 | 0.8079 | [M-H]- |
| 9 | 8.22 | morin | C_15_H_10_O_7_ | 301.0354 | 301.0359 | 1.7410 | [M-H]- |
| 10 | 8.58 | isoorientin | C_21_H_20_O_11_ | 447.0933 | 447.0931 | 1.1528 | [M-H]- |
| 11 | 8.58 | astragalin | C_21_H_20_O_11_ | 447.0933 | 447.0938 | 1.1528 | [M-H]- |
| 12 | 8.58 | luteolin-7-o-glucoside | C_21_H_20_O_11_ | 447.0933 | 447.0938 | 0.4129 | [M-H]- |
| 13 | 10.13 | quercetin | C_15_H_10_O_7_ | 301.0354 | 301.0354 | 0.0000 | [M-H]- |
| 14 | 10.21 | kaempferol | C_15_H_10_O_6_ | 285.0405 | 285.0406 | 0.4866 | [M-H]- |
| 15 | 11.73 | EIC | C_18_H_32_O_2_ | 279.2330 | 279.2330 | 0.0000 | [M-H]- |
| 16 | 13.55 | malvidin | C_17_H_15_ClO_7_ | 330.0745 | 330.0732 | 3.9415 | [M-H]- |
| 17 | 14.85 | lauric acid | C_12_H_24_O_2_ | 199.1704 | 199.1698 | 2.7780 | [M-H]- |
| 18 | 14.88 | ethyl caffeate | C_11_H_12_O_4_ | 207.0663 | 207.0657 | 2.8112 | [M-H]- |
| 19 | 16.31 | hyperin | C_21_H_20_O_12_ | 463.0882 | 463.0886 | 0.8655 | [M-H]- |
| 20 | 16.39 | myristic acid | C_14_H_28_O_2_ | 227.2017 | 227.2013 | 1.5554 | [M-H]- |
| 21 | 16.61 | ursolic acid | C_30_H_48_O_3_ | 455.3531 | 455.3534 | 0.7278 | [M-H]- |
| 22 | 18.05 | oleic acid | C_18_H_34_O_2_ | 281.2486 | 281.2487 | 0.3428 | [M-H]- |
| 23 | 18.23 | palmitic acid | C_16_H_32_O_2_ | 255.2330 | 255.2327 | 0.9932 | [M-H]- |
| 24 | 18.98 | stearic acid | C_18_H_36_O_2_ | 283.2643 | 283.2643 | 0.0000 | [M-H]- |
| 25 | 19.61 | montanic acid | C_28_H_56_O_2_ | 423.4208 | 423.4209 | 0.3441 | [M-H]- |
| 26 | 21.67 | lignoceric acid | C_24_H_48_O_2_ | 367.3582 | 367.3584 | 0.6696 | [M-H]- |
| 27 | 24.28 | esculetin | C_9_H_6_O_4_ | 177.0193 | 177.0186 | 4.1351 | [M-H]- |

Table S2 Identification results of chemical compounds in the TH sample

| Num | t_R_/min | Identified results | Formula | *m/z* theoretical | *m/z* measured | Error/  ppm | Detection mode |
| --- | --- | --- | --- | --- | --- | --- | --- |
|  |  |  |  |  |  |  |  |
| 1 | 0.79 | cichoric acid | C_22_H_18_O_12_ | 473.0725 | 473.0728 | 0.6342 | [M-H]- |
| 2 | 2.05 | caffeic acid | C_9_H_8_O_4_ | 179.0350 | 179.0343 | 3.9099 | [M-H]- |
| 3 | 2.53 | 4-hydroxy-3-methoxybenzaldehyde | C_8_H_8_O_3_ | 151.0401 | 151.0391 | 6.6208 | [M-H]- |
| 4 | 2.90 | chlorogenic acid | C_16_H_18_O_9_ | 353.0878 | 353.0880 | 0.5664 | [M-H]- |
| 5 | 3.45 | benzenecarboxylic acid | C_7_H_6_O_2_ | 121.0295 | 121.0284 | 9.1110 | [M-H]- |
| 6 | 4.17 | p-coumaric acid | C_9_H_8_O_3_ | 163.0401 | 163.0392 | 5.5201 | [M-H]- |
| 7 | 4.53 | protocatechuic aldehyde | C_7_H_6_O_3_ | 137.0244 | 137.0234 | 7.2980 | [M-H]- |
| 8 | 7.62 | isochlorogenic acid A | C_25_H_24_O_12_ | 515.1195 | 515.1199 | 0.7765 | [M-H]- |
| 9 | 9.21 | 3,4-dicaffeoylquinic acid | C_25_H_24_O_12_ | 515.1195 | 515.1199 | 0.7765 | [M-H]- |
| 10 | 9.77 | rutin | C_27_H_30_O_16_ | 609.1461 | 609.1469 | 1.3133 | [M-H]- |
| 11 | 9.77 | quercetin-3-o-galactoside | C_21_H_20_O_12_ | 463.0882 | 463.0887 | 1.0797 | [M-H]- |
| 12 | 10.74 | luteolin-7-o-glucoside | C_21_H_20_O_11_ | 447.0933 | 447.0936 | 0.6710 | [M-H]- |
| 13 | 11.20 | arsanin | C_15_H_22_O_4_ | 265.1445 | 265.1444 | 0.3772 | [M-H]- |
| 14 | 11.92 | taraxinic acid 1'-o-beta-d-glucopyranoside | C_21_H_28_O_9_ | 423.1661 | 423.1637 | 5.6715 | [M-H]- |
| 15 | 13.58 | quercetin | C_15_H_10_O_7_ | 301.0354 | 301.0355 | 0.3322 | [M-H]- |
| 16 | 13.90 | ethyl caffeate | C_11_H_12_O_4_ | 207.0663 | 207.0658 | 2.4147 | [M-H]- |
| 17 | 14.73 | apigenin | C_15_H_10_O_5_ | 269.0455 | 269.0455 | 0.0000 | [M-H]- |
| 18 | 23.20 | luteolin | C_15_H_10_O_6_ | 285.0405 | 285.0406 | 0.3508 | [M-H]- |
| 19 | 23.38 | myristic acid | C_14_H_28_O_2_ | 227.2017 | 227.2012 | 2.2007 | [M-H]- |
| 20 | 24.33 | palmitic acid | C_16_H_32_O_2_ | 255.2330 | 255.2328 | 0.7836 | [M-H]- |
| 21 | 25.11 | stearic acid | C_18_H_36_O_2_ | 283.2643 | 283.2644 | 0.3530 | [M-H]- |

Table S3 Content determination results of 8 compounds in PVL from different origins

| Extraction solvent | compound | Content (mg/g) | | | | | |
| --- | --- | --- | --- | --- | --- | --- | --- |
|  |  | Henan  (20210822) | Hubei  (20210906) | Jiangsu  (20210926) | Sichuan  (20220111) | Anhui  (20220103) |  |
| Water | Caffeic acid | 0.802 | 0.981 | 0.535 | 0.666 | 1.021 |  |
|  | P-coumaric acid | 0.044 | 0.035 | 0.043 | 0.034 | 0.026 |  |
|  | Scopoletin | - | - | - | - | - |  |
|  | Rosmarinic acid | 1.097 | 1.124 | 0.685 | 1.545 | 1.766 |  |
|  | Esculetin | 0.040 | 0.028 | 0.027 | 0.046 | 0.037 |  |
|  | Rutin | 0.037 | 0.027 | 0.013 | 0.028 | 0.043 |  |
|  | Hyperoside | - | - | - | - | - |  |
|  | Quercetin | 0.041 | 0.039 | 0.042 | 0.028 | 0.021 |  |
| 50% ethanol | Caffeic acid | 0.301 | 0.326 | 0.237 | 0.273 | 0.361 |  |
|  | P-coumaric acid | 0.021 | 0.017 | 0.021 | 0.016 | 0.013 |  |
|  | Scopoletin | 0.035 | 0.051 | 0.098 | 0.037 | 0.044 |  |
|  | Rosmarinic acid | 3.389 | 3.874 | 1.590 | 4.392 | 5.240 |  |
|  | Esculetin | 0.037 | 0.026 | 0.026 | 0.041 | 0.033 |  |
|  | Rutin | 0.306 | 0.236 | 0.110 | 0.241 | 0.368 |  |
|  | Hyperoside | 0.157 | 0.126 | 0.077 | 0.134 | 0.195 |  |
|  | Quercetin | 0.025 | 0.020 | 0.026 | 0.015 | 0.010 |  |

Table S4 Content determination results of 6 compounds in TH from different origins

| Extraction solvents | compound | Content (mg/g) | | | | | |
| --- | --- | --- | --- | --- | --- | --- | --- |
|  |  | Henan  (20210814) | Anhui  (20210925) | Shanxi  (20210510) | Gansu  (20210522) | Hunan  (20210826) |  |
| Water | Chlorogenic acid | 0.681 | 0.352 | 0.522 | 0.423 | 0.920 |  |
|  | Cichoric acid | 3.334 | 1.904 | 3.221 | 3.007 | 4.236 |  |
|  | Caffeic acid | 0.183 | 0.125 | 0.869 | 0.855 | 0.803 |  |
|  | Luteolin | 0.067 | 0.035 | 0.036 | 0.042 | 0.030 |  |
|  | P-coumaric acid | 0.074 | 0.031 | 0.122 | 0.074 | 0.075 |  |
|  | Rutin | 0.079 | 0.049 | 0.047 | 0.058 | 0.037 |  |
| 50% ethanol | Chlorogenic acid | 0.471 | 0.265 | 0.421 | 0.309 | 0.636 |  |
|  | Cichoric acid | 2.284 | 1.701 | 2.407 | 1.824 | 2.964 |  |
|  | Caffeic acid | 0.231 | 0.199 | 0.531 | 0.480 | 0.445 |  |
|  | Luteolin | 0.314 | 0.183 | 0.188 | 0.216 | 0.138 |  |
|  | P-coumaric acid | 0.045 | 0.021 | 0.042 | 0.039 | 0.042 |  |
|  | Rutin | 0.209 | 0.177 | 0.149 | 0.188 | 0.136 |  |
